# Neuroethics 1995–2012. A Bibliometric Analysis of the Guiding Themes of an Emerging Research Field

**DOI:** 10.3389/fnhum.2016.00336

**Published:** 2016-07-01

**Authors:** Jon Leefmann, Clement Levallois, Elisabeth Hildt

**Affiliations:** ^1^Neuroethics Research Group, Department of Philosophy, Johannes Gutenberg-UniversitätMainz, Germany; ^2^Department of Markets and Innovation, EMLYON Business SchoolÉcully, France; ^3^Center for the Study of Ethics in the Professions, Illinois Institute of TechnologyChicago, IL, USA

**Keywords:** history of neuroethics, neuroethics, scientometrics, bibliography, science studies, Mainz Neuroethics Database

## Abstract

In bioethics, the first decade of the twenty-first century was characterized by the emergence of interest in the ethical, legal, and social aspects of neuroscience research. At the same time an ongoing extension of the topics and phenomena addressed by neuroscientists was observed alongside its rise as one of the leading disciplines in the biomedical science. One of these phenomena addressed by neuroscientists and moral psychologists was the neural processes involved in moral decision-making. Today both strands of research are often addressed under the label of neuroethics. To understand this development we recalled literature from 1995 to 2012 stored in the Mainz Neuroethics Database (i) to investigate the quantitative development of scientific publications in neuroethics; (ii) to explore changes in the topics of neuroethics research within the defined time interval; (iii) to illustrate the interdependence of different research topics within the neuroethics literature; (iv) to show the development of the distribution of neuroethics research on peer-reviewed journals; and (v) to display the academic background and affiliations of neuroethics researchers. Our analysis exposes that there has been a demonstrative increase of neuroethics research while the issues addressed under this label had mostly been present before the establishment of the field. We show that the research on the ethical, legal and social aspects of neuroscience research is hardly related to neuroscience research on moral decision-making and that the academic backgrounds and affiliations of many neuroethics researchers speak for a very close entanglement of neuroscience and neuroethics. As our article suggests that after more than one decade there still is no dominant agenda for the future of neuroethics research, it calls for more reflection about the theoretical underpinnings and prospects to establish neuroethics as a marked-off research field distinct from neuroscience and the diverse branches of bioethics.

## Introduction

Ethical questions related to the treatment of those, who suffer from neurological and mental illness, have been at issue in one or the other way since the beginning of medicine (Berrios, 1995). Similarly, also the scientific investigation into the psychological and biological determinants of human behavior in morally relevant contexts has a long history, which has sometimes yielded disturbing and sometimes even questionable results (Schleim, 2009; Schirmann, 2013). However, it was only with the rise of neuroscience as one of the leading scientific disciplines of the twenty-first century and the simultaneous development of new technology to investigate and control brain mechanisms that scientists, philosophers and bioethicists saw the need for a field of research and reflection they called neuroethics. Usually the beginning of neuroethics as a discipline is regarded as linked to a series of interdisciplinary conferences bringing together neuroscientists, philosophers, policymakers and bioethicists that took place in several places in the UK and the USA (Roskies, 2002; Moreno, 2003). In particular, the conference “Neuroethics: Mapping the Field” held with financial support of the Dana foundation in May 2002 in San Francisco received widespread attention (Marcus, 2002). Even though the rapid development of knowledge on the brain and the biology of the human mind made a systematic approach toward its ethical, legal, and social consequences exceptionally pressing at that time, there is evidence that the label “neuroethics” appeared in the contexts of neuroscience, medical ethics and philosophy of mind long before 2002 (Pontius, 1973, 1993; Cranford, 1989; Churchland, 1991). This suggests that what is dubbed “neuroethics” today has historical progenitors in debates from very different disciplinary fields. In this perspective, neuroethics has emerged as an interdisciplinary endeavor connecting substantively and methodologically diverse scientific and philosophical approaches only in subsequence of the growing knowledge in neuroscience and the associated promise of linking the subjective and personal world of experience and thought to the objective world of scientific data. Because of this, we conceive of neuroethics as a developing research field and not as an established discipline with clearly defined goals and methods. This characterization as a juvenile and emerging field of research has made the demarcation of neuroethics from adjoining disciplines an important issue ever since the field's official birth (Roskies, 2002; Cabrera, 2011; Levy, 2011).

As philosophers have learned from longstanding debates in the philosophy of science, the demarcation of a scientific research field is partly a prescriptive and stipulative issue and partly a descriptive and explanatory issue (Laudan, 1983; Resnik, 2000). This also holds true for approaches to mark off neuroethics from its neighboring fields of research. A good definition of a research field not only prescribes which questions are central and which do not belong to it, a good definition also needs to track the phenomenon as it factually is; at least to a certain degree. A definition of neuroethics that did not offer a criterion to distinguish between issues being relevant or irrelevant to the field, would not be a definition at all. Equally a definition, which would not relate to the scientific literature published under that label or that would not—to a certain extent—relate to the currently held understanding of those working in the field, would not be a definition of the phenomenon in question, but a definition of something else.

The variety of conceptualizations of neuroethics, which have been proposed since 2002, suggests that defining neuroethics has been primarily an issue of adequately marking off the field from other branches of bioethics on the one hand and from neuroscience on the other hand. As a first mapping of such definitional approaches, Eric Racine has suggested that we distinguish between technology-driven, healthcare-driven and knowledge-driven definitions of neuroethics (Racine, 2010). Focusing primarily on the applications of new technologies that affect the mind and the brain cognitive scientist Martha J. Farah, for example, promoted a technology-driven and comparatively narrow concept of neuroethics as social, legal and ethical implications of cognitive neuroscience (Farah, 2007). From a point of view strongly influenced by clinical neuroscience, Racine and Illes propose a health-care driven definition that conceptualizes neuroethics as a field “at the intersection of neuroscience and bioethics defined by a general practical goal, that of improving patient care for specific patient populations” (Racine and Illes, 2009). Yet another kind of definition is strongly driven by the interest of ascribing empirical knowledge from neuroscience and psychology an important role in neuroethics (Roskies, 2002; Evers, 2005; Levy, 2007, 2011). The by now classic division of neuroethics into the “ethics of neuroscience” and the “neuroscience of ethics” (Roskies, 2002) belongs to this strand of knowledge-driven conceptualizations. Authors following these kinds of definitions conceive of neuroethics not only as a normative endeavor in applied bioethics (ethics of neuroscience), but propose to varying extents that knowledge from neuroscience can or even ought to shape “our understanding of ethics itself” (neuroscience of ethics) (Levy, 2007). Different definitions including empirical neuroscience as relevant to neuroethics have been proposed by Giordano^1^ and Scott (Scott, 2012) and have most radically been advanced by psychologist Michael Gazzaniga, who envisions a general “philosophy of living informed by our understanding of underlying brain mechanisms” (Gazzaniga, 2005) and philosopher Patricia Churchland (Churchland, 2011). Expanding the concept of neuroethics into the area of empirical knowledge about brain mechanisms involved in human behavior in moral contexts, however, made it necessary to reflect on the basic theoretical framework of the research field (Evers, 2007; Northoff, 2009). Ascribing relevance to neuroscience for the normative questions addressed in ethics requires an account about how the descriptive propositions produced in neuroscience could inform the normative propositions made in ethics. This paved the way for a strand of metaethical discussions with relation to neuroethics (Sinnott-Armstrong, 2006; Joyce, 2008; Kennett and Fine, 2008), which, however, are only seldom included into the research agenda of the field (Racine, 2010; Wagner and Northoff, 2015).

With this study, we aim to complement the ongoing process of conceptualizing neuroethics as a research field with new empirical data from the Mainz Neuroethics Database (MND). Drawing on a large corpus of the scientific literature from the last quarter century, we are interested in two different aspects of neuroethics: First, its development into an institutionalized research field distinct from neuroscience and the diverse branches of biomedical ethics, and second the relations between the seemingly heterogeneous research questions within the field. To understand the current structure and development of neuroethics, we focus on the years between 1995 and 2012 when neuroethics slowly begins to become visible at the margins of the established disciplines of neuroscience, bioethics, medical ethics and the philosophy of mind, and starts to develop into a widely observed research field represented by particular scientific journals and research facilities. Using bibliometric analysis, we (i) investigate the quantitative development of scientific publications in neuroethics, (ii) explore changes in the topics and guiding themes of neuroethics research, (iii) illustrate the interdependence of different research topics within the neuroethics literature, (iv) demonstrate the development of the distribution of neuroethics research in peer-reviewed journals, and (iv) display the academic background and affiliations of neuroethics researchers. These investigations will help to highlight the main differences and similarities of neuroethics and adjoining scientific disciplines and contribute to a better understanding of the current state of neuroethics. Previous bibliometric work on this field has drawn on much smaller subsets of literature (Racine, 2010; Gooray and Ferguson, 2013), has addressed only very particular research fields within the much broader spectrum of neuroethics literature (Seixas and Ayres Basto, 2008; Lombera and Illes, 2009; Garnett et al., 2011, 2013; Boelsen, 2016) or has provided structured compilations of neuroethics literature without any quantitative further analysis (Buniak et al., 2014; Darragh et al., 2015). With this paper we offer the first comprehensive empirical analysis of the development and current state of neuroethics.

## Methods

### The Mainz Neuroethics Database

Our empirical analysis draws on the scientific literature stored in the Mainz Neuroethics Database (MND) (available at https://teamweb.uni-mainz.de/fb05/Neuroethics/Lists/Bibliography/Show.aspx). The MND started as an open-access online bibliography in 2006. It resulted from a multimodal compilation of publications (journal articles, books, edited volumes etc.) in various languages, which referred to the term “neuroethics” or the respective equivalent non-English word or which were considered to be relevant to the field. Currently the database contains more than 4000 publications in English, French, German, Italian, Japanese and Spanish that appeared between 1975 and 2015. The members of the research group searched for publications to add to the database by regular scans of relevant journals from the neurosciences as well as from philosophy and other humanities and social sciences, by regularly searching the PubMed and Scopus databases and by making use of bibliographic thematically relevant literature lists (i.e., Brainstorm Newsletter). The literature added to the database was not restricted to research articles but also included editorials, opinion papers and reviews. Anthologies with an obvious reference to the neuroethics discourse were also added to the database. For the selection of publications from the various sources, the following criteria were used: Publications appearing in journals from the empirical neurosciences needed to make a reference to ethical or social impacts of the presented results, whereas publications appearing in journals from the humanities or social sciences needed to refer to empirical results from neuroscience or from psychological research. Furthermore, publications, which did not display this kind of transdisciplinarity were added if they were considered to be relevant to the field. For example, a paper presenting empirical work on memory functions was added to the database, if it considered the possibility to technically manipulate memory or explicitly discussed the issue of intentional memory modification. Likewise, a philosophical paper on the structure of the human mind was included, if it referred for argumentative purposes to studies on the brain processes underlying decision-making and perception in moral contexts.

### Eligibility criteria and bibliometric method

To allow for bibliometric analysis of the versatile material stored in the MND, we first performed a series of heuristics and manual checks to disambiguate author names present in several orthographical variations (for example, Baumeister, Roy and Baumeister, Roy F are merged into a single author name). This ensured a consistent identification and count of authors across the database and lowered the number of relevant publications. Second, we restricted the data corpus to articles that appeared between 1995 and 2012—a time interval that centers around the year of the founding conferences of current neuroethics and which therefore appeared most relevant for monitoring the development of the field. This left us with a corpus of 2296 relevant references. Third, publications without an English abstract and keywords were sorted out, as they did not provide enough information for our procedure of automatic thematic categorization of publications. This step excluded the bulk of editorials and opinion papers as well as most of the non-peer-reviewed material from the dataset, 371 publications (16.16%). Forth, each of the remaining 1925 publications was categorized according to a set of topical subject-categories (cf. Section Subject-Category Development and Supplements 1A, B in the Supplementary Material). For classification, the text of the abstract, headline and keywords of the publication were examined for matches with keywords associated with topical subject-categories. In case of a match, the publication was tagged with the subject-category corresponding to the matching keyword. For example, a reference containing the keyword “cosmetic neurology” was tagged with the “Enhancement” subject-category (for a list of keywords see Supplement 1B in the Supplementary Material). It required at least two matching keywords to tag a reference as falling under a subject-category. References were allowed to be tagged as belonging to more than one subject-category, allowing for analysis of pointwise mutual information (PMI) with respect to subject categories (cf. Section Connections between Subject-Categories). Finally, the available information extracted from all references was aggregated and sorted according to (i) frequency of subject-categories per year, (ii) frequency of authors per subject-category, (iii) frequency of journals per subject-category, and (iv) connections between subject-categories.

### Subject-category development

Subject-categories were created using the three subsequent steps of coding, identification of relevant keywords defining the subject-categories, and checks for the reliability of keyword matches.

#### Coding

Based on the headlines and abstracts, 400 randomly retrieved publications from the dataset were coded according to the topics they addressed. The coding was done independently using a close reading approach by EH and JL, who are both experts in the field of neuroethics. The coding was reviewed for reliability by another research assistant. By careful evaluation of abstracts, headlines and keywords of the bibliographical references, this procedure allowed for development of a system of 44 subject-categories. Candidates for subject-categories were general topics addressed in these text components. For example, “Enhancement” and “neurodegenerative diseases” were suitable candidates for such general topics, because both occurred in the literature with regard to several specific contexts, such as “pharmacological cognitive enhancement” and “brain-stimulation” or “Alzheimer's disease” and “aging” respectively. As the number of publications per subject-category strongly varied after coding, similar subject-categories, which contained less than five publications were merged into a new, more general subject-category. This slightly reduced the number of subject-categories from 44 to 38.

#### Identification of relevant keywords

The second step was conducted together with CL, an expert in both neuro-social sciences and the methodology of computational linguistics, and aimed at defining a list of keywords for each subject-category to allow for an automated matching of publications with subject-categories. This step comprised itself five operations (c.f. Supplement 1A in the Supplementary Material): In the first operation we identified five intuitive and consensual keywords retrieved from the grouped publications for each subject-category. For example, the category “addiction” was identified by the keywords “addict,” “abuse,” “compulsion,” “craving,” and “dependence.” The second operation was an automatic search for these consensual keywords in the headlines, abstracts and keywords in all publications from the time interval 1995–2012. In a third operation, additional terms related to the topic of each subject-category were identified by close reading of a random samples of 15 publications that were attributed to the respective subject-category in the automatic search. For example, in addition to the consensual keywords defined in step one, papers with the topic “addiction” revealed terms used specifically in relation to addiction, such as “alcohol” or different forms of the root “addict,” such as “addicted” or “addicts,” which we added to the defining keyword-list of the subject category. The fourth operation merged categories, which shared more than half of their keywords, when all authors agreed after having discussed the issue drawing on their expertise in neuroethics. Finally, in the fifth operation, papers were again categorized according to the extended keyword list, and the operations one to five were repeated until no further plausible keywords for each of the topics could be identified. Repetition of the operations one to five resulted in a system of 15 final subject-categories (c.f. Table 1). To allow for even greater specificity of the keyword list, we additionally refined the matching of publications with subject-categories by defining a threshold for one or several keywords from the final keyword-lists of some of the categories. This allowed us to account for the differing degrees of semantic specificity of some of the keywords. For example we found that if the term “neuroimaging” was present in an abstract, it identified a paper as belonging to the subject-category “Neuroimaging” with a high degree of certainty (for a quantification of this degree cf. Supplement 1D in the Supplementary Material). At the same time, however, the keyword “public” alone did not point to a specific topic, while co-occurrence with the term “policy” reliably points to the topic “(Neuro)science and society.” Hence, we allowed papers to be tagged in the subject-category “Neuroimaging” based on the inclusion of a single, very specific keyword while a classification in the subject-category “(Neuro)science and society” necessitated at least co-occurrence of two keywords. This procedure has no in-built logic to guarantee that each topic will be defined by a constant number of keywords. On the contrary, in our approach the specificities of the vocabulary in each field determine the length of the keyword-lists. Hence the number of keywords defining a subject-category varies between 8 and 27 in our approach. We believe, however, that the specificity of the vocabulary used in the final keyword-lists accounts much better for the topical character of the papers than an approach based on a constant quantity of keywords per category. We further discuss this methodological point in the Section on Limitations.

**Table 1:** **Distribution of subject-categories over journals and articles from the MND**.

**Subject-category**	**Total number of journals**	**Share of biomedical science journals in %**	**Share of SSH journals in %**	**Total number of articles**	**Share of articles in biomedical science journals in %**	**Share of articles in SSH Journals in %**
Psychiatric and neurodegenerative diseases and disorders	49	69.39	30.61	342	64.33	35.67
Neuroimaging	47	74.47	25.53	279	70.61	29.39
Moral theory	30	56.67	43.33	181	43.09	56.91
Philosophy of mind and consciousness	28	42.86	57.14	155	38.71	61.29
Medical research and medicine	24	50	50	137	37.23	62.77
Neurosurgery	22	59.09	40.91	115	40.87	59.13
Legal studies	19	36.84	63.16	108	27.78	72.22
Neuroscience and society	14	35.71	64.29	81	34.57	65.43
Psychopharmacology	18	66.67	33.33	88	50	50
Social and economic neuroscience	18	72.22	27.78	76	75	25
Brain death/severe disorders of consciousness	14	57.14	42.86	82	46.34	53.66
Brain stimulation	13	61.54	38.46	65	53.85	46.15
Enhancement	11	45.45	54.55	59	32.20	67.80
Molecular neurobiology and genetics	2	50.00	50.00	6	50.00	50.00
Addiction	4	25.00	75.00	19	15.79	84.21

#### Reliability checks

To check whether the keywords defining the 15 subject-categories were able to cover all the relevant topics of the publications in the database and to control for the adequacy of computer-based assignments of subject-categories, we manually checked the computer-based category assignments of 100 randomly selected publications. Our control suggested an inadequate assignment of categories in only 12% of the articles, a measure, which we consider still sufficiently accurate. To verify that the content of the bibliographical references harvested in each topic was indeed in line with the topic, we additionally analyzed the terms used in the abstracts, titles and keywords of the publication, which were assigned to a subject-category and evaluated wether they matched with the topic of the subject-category. This was achieved by using a “bag of words”-model, which implies counting all terms included in the bibliographical reference categorized as matching a subject-category and using this count as a quantifier for the relevance of a term relative to the subject-category. As the most common terms for all categories would usually be common expressions like “this study shows” or “we conclude,” in a first step of word-bag processing we removed general stopwords (such as “a,” “the,” “have”) and stopwords specific to the scientific domain (such as “we find,” “the hypothesis tested,” “we conclude” etc.). Then, we calculated the term frequency-inverse document frequency (tf-idf) for each term by discounting the frequency of a given term in a subject-category from its frequency in all bibliographical references across subject-categories. This takes into account that a term is all the more descriptive of a subject-category, in which it is specifically used. For example, a term used many times in the bibliographical references classified in the category “Neuroimaging,” which is never used in bibliographical references assigned to other categories, has a high tf-idf score in the category “Neuroimaging.” The results of this reliability check are presented in Supplement 5 in the Supplementary Material, where we list the 25 terms with the highest tf-idf per subject-category. This data shows that there is a strong semantic proximity between the terms with the highest tf-idf of a given topic and the label of this topic. This confirms that the automated categorization procedure yielded consistent results.

## Results

### The quantitative development of neuroethics since 1995

To estimate the development of neuroethics as a discipline we first looked at the quantitative development of published work in the field. Looking at the distribution of publication dates, we found a general increase in publications within the selected time interval. While only eight publications with references to neuroethics have appeared in 1995 (0.35% of all publications) in 2009, there were already 590 publications (25.7%) in the database. Within the examined dataset, the increase in the number of publications was continuous except for the interval between 2009 and 2012. While in this interval the number of publications per year remained on a comparably high level, instead of increasing any further the number of publications per year fluctuated between 145 (6.32%) in 2010 and 348 (15.16%) in 2011. This striking fluctuation does not represent a feature of the development of neuroethics, but is very likely an artifact created through the maintenance of the MND. A closer analysis of the publications added to the database in the years 2009 and 2010 revealed discrepancies in the number of journals that contributed paper to the database (274 in 2009 and 83 in 2010) and the number of monographs and book chapters included. As our data show, this variety concerns all subject-categories and can only partly be explained by the founding of the journal “Neuroethics” and “AJOB Neuroscience” in 2008 and 2010 respectively. It is more likely that it relies on inconsistent in- and exclusions of publications in the years 2009 and 2010.

**Figure 1:**
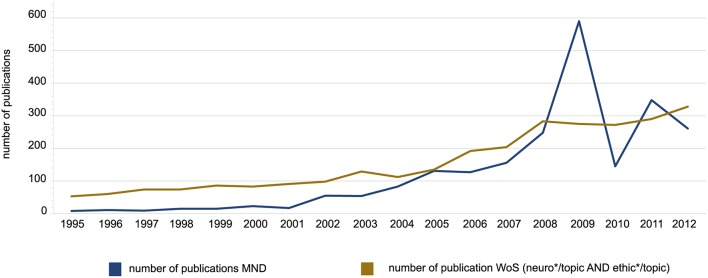
**Development of the number of annual publications in neuroethics according to MND and Worlds of Science (WoS)**. It is apparent that publications in neuroethics have rapidly increased in number particularly after the year 2002. The x-axis describes the year in which the publications appeared the y-axis describes the number of papers published each year. The blue line describes the development according to the dataset retrieved from the MND, the brown line according to the dataset retrieved from the Web of Science (WoS) database.

The general trend of the temporal development of publication numbers in the Mainz database is consistent with the development of the number of publications in other databases such as Web of Science (WoS). We tried several protocols for searches in the WoS database with only one protocol (neuro^*^/topic AND ethic^*^/topic) providing an approximately similar number and distribution of articles per year. In both databases there is a moderate increase in the number of publications between 1995 and 2005 (+8.3% in MND and +6.5% in WoS), a stronger increase between 2005 and 2009 (+101.4% and +32.6% respectively) and an inconsistent development between 2009 and 2012 (−109.7% and +17.7%) (cf. Figure 1). Moreover there is a similar overall increase across the whole time-span in both databases (+14.6% in MND and +16.7% in WoS). Except for the years 2009 and 2011 there is a higher total number of publications in the WoS database than in the MND, which is due to the more specific selection of publications in the MND. The data from WoS suggests a stable plateau for the number of publications after 2009.

### Quantitative description of the subject-categories

**Figure 2:**
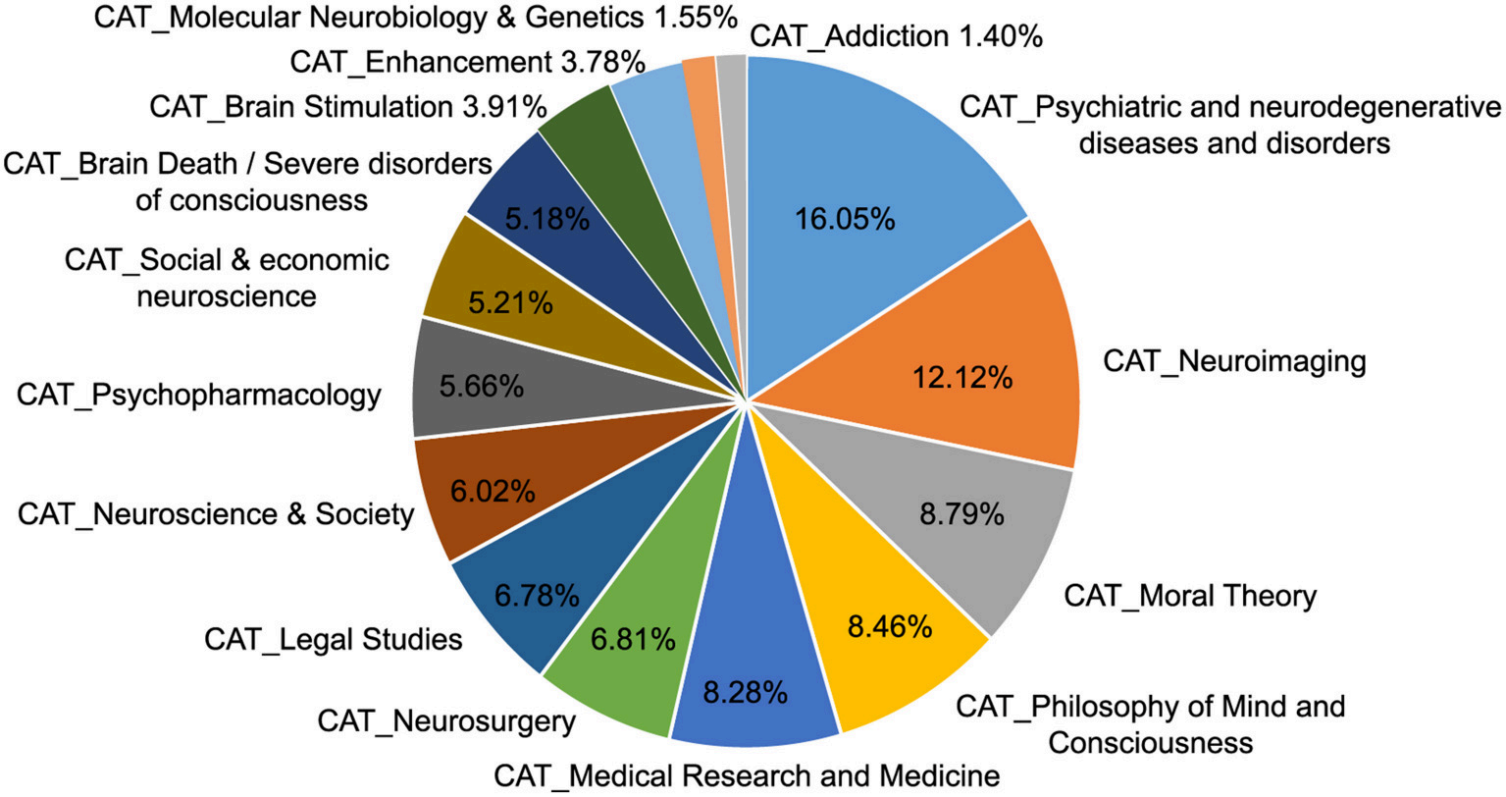
**Size of thematic subject-categories in the MND**. The pie chart illustrates the size of the different thematic categories that form the research field of neuroethics. The categories were derived from headlines, abstracts and keywords of the publications via an intersubjective and iterative coding procedure. The size of the slices and the corresponding numbers indicate the number of publications that were tagged as belonging to the respective category.

The procedure of subject-category formation provided a structure, which divided the content of the database into 15 subject-categories (see Supplement 1 in the Supplementary Material). Of the 2296 publications retrieved from the MND, 1925 publications could be assigned at least one subject-category. Most of these publications belonged to more than one subject-category with an average number of 1.7 subject-category attributions per publication. To assess the distribution of research topics in neuroethics during the last quarter century we counted the number of publications tagged in each of the categories (cf. Figure 2). The largest share of publications (14.67%) was tagged as belonging to the subject-category *Psychiatric and neurodegenerative diseases and disorders*, the smallest share (1.28%) belonged to the subject-category *Addiction*. There is a large majority of publications (73.17%), which are tagged as part of subject-categories primarily associated with ethical, legal and social aspects of neuroscience (*Psychiatric and neurodegenerative diseases and disorders, (Medical) research and medicine, Legal Studies, Neuroscience and Society, Psychopharmacology, Social and economic neuroscience, Brain death and severe disorders of consciousness, Brain Stimulation, Molecular Neurobiology and Genetics, Addiction*), whereas only a minority of articles refers to aspects associated with neuroscience research about reasoning and decision-making in social and moral contexts (*Moral theory, Philosophy of mind an consciousness, Neuroimaging*). For a precise and content-based description of the subject-categories including examples see also Supplement 1C in the Supplementary Material.

### Development of subject-categories between 1995 and 2012

**Figure 3:**
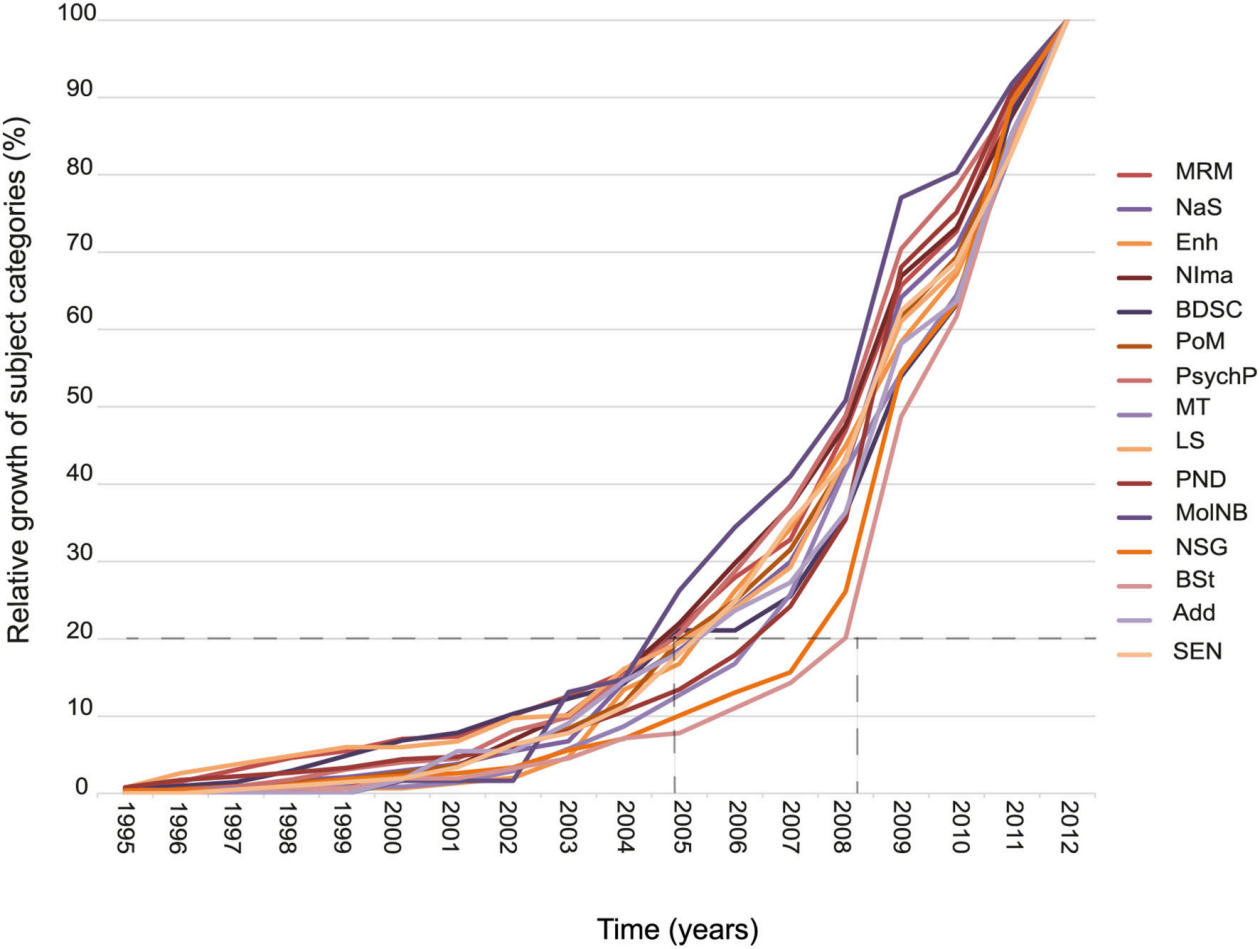
**Development of annual publications within thematic subject-categories**. The diagram displays the growth of the number of publication per subject-category relative to the overall number of publications per subject-category in the interval 1995–2012 (baseline = 100%). The dashed lines indicate variation of growth between different subject-categories. Whilst 20% of all publications in the category *Neuroimaging* (*NIma*, dark red line) appeared before 2005, it took until 2008 for 20% of all publications in the category of *Brain Stimulation* (*BSt*, flesh tinted line) to appear.

There is a general trend in all subject-categories of an increasing number of publications. With only few exceptions most of the publications within one subject-category appeared after 2005 (for details see below). In spite of some variations (for example in the relative weight of the subject-category “*Psychopharmacology*” or “*Social and economic neuroscience*”), over the years the relative shares of the subject-categories remained relatively constant (see Figure 3). To assess the slope of the increase within each subject-category we determined the year until 20% of the articles within a subject-category had been published. Usually this threshold was reached in the year 2005 (holds for *Brain death/Severe disorders of consciousness, Medical Research and medicine, Philosophy of mind and consciousness, Molecular Neurobiology and Genetics, Legal Studies, Neuroimaging, Psychopharmacology and Neuroscience and society*). Only in *Enhancement* was the threshold reached earlier (2004), while in the remaining subject-categories it was only reached between 2006 and 2008.

### Connections between subject-categories

**Figure 4:**
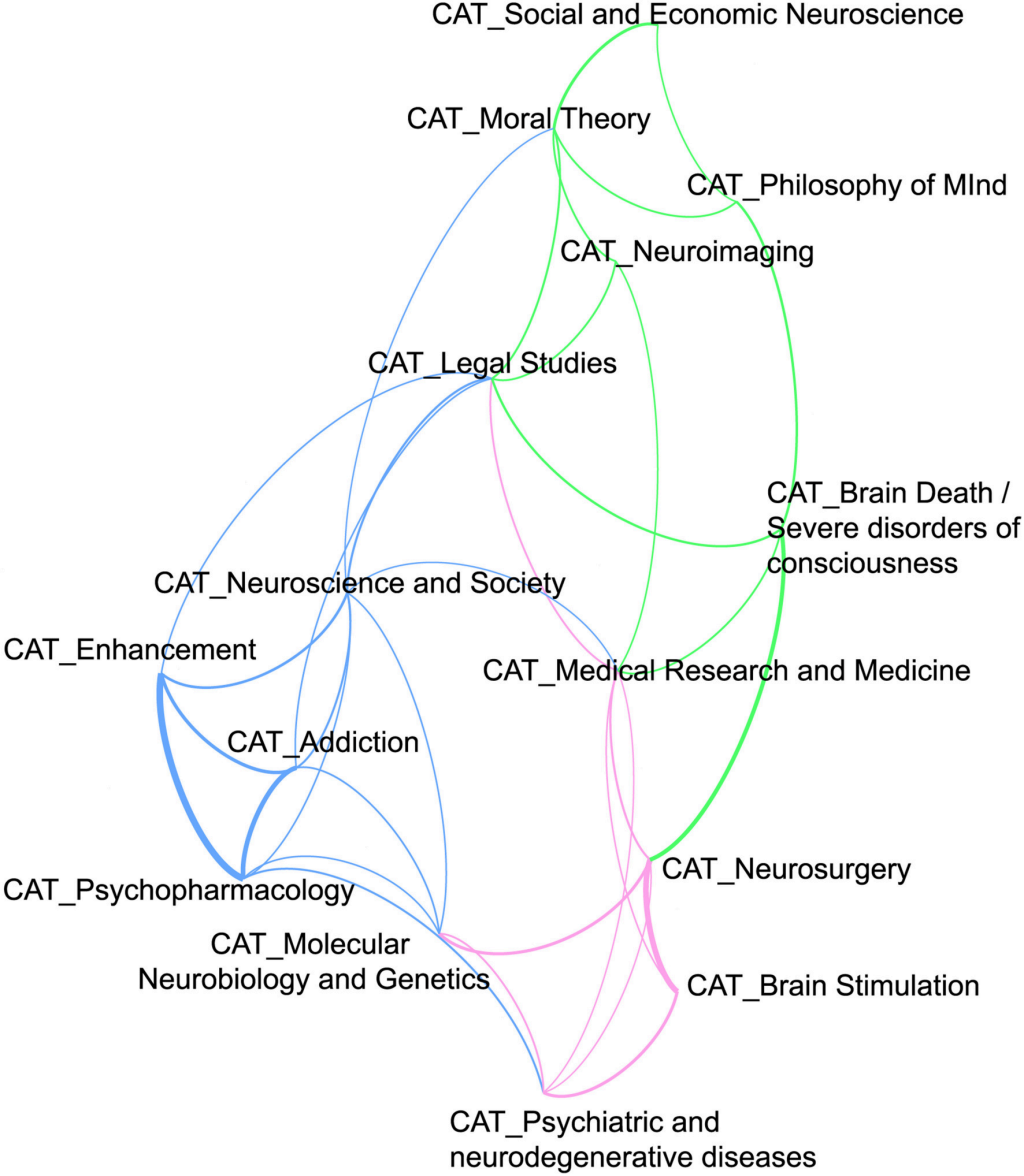
**Thematic map of neuroethics as a research field**. The figure maps the relation of the different subject-categories measured by pointwise mutual information (PMI). Only relations of subject-categories with a PMI of 0.7 or higher are displayed. The thicker a connecting line, the higher the PMI. The length of the connecting lines is irrelevant.

To further investigate the relation between the different subdomains of neuroethics, we determined how subject-categories relate to each other from a content-based point of view. To assess the strength of the connections between two subject-categories, we determined their pointwise mutual information (*i*_*a, b*_) (Islam and Inkpen, 2006). I_*a, b*_ quantifies the discrepancy between the probability of the coincidence of subject-categories being attributed to a publication and the probability of their individual attribution. Additionally, we accounted for the higher informational relevance of connections between rare topics by applying a discount factor to *i*_*a, b*_. This higher informational relevance is explained by the fact that the co-occurrence of rare topics is comparatively much more unlikely as that of less rare topics. To apply this discount factor, we divided the number of papers sharing two topics by the product of the number of papers per subject-category (for a formular cf. Supplement 1E in the Supplementary Material). This is a close variant of the pointwise mutual information measure commonly used in computational linguistics. A map showing the 15 subject-categories connected by colored lines representing discounted *i*_*a, b*_ measure above 0.7 (cf. Figure 4) functions as a visual tool displaying the correlations between topics in the neuroethics literature.

We found three groups of closely interrelated subject-categories. The first group comprises literature associated with topics in clinical medical ethics (*Brain Death and severe disorders of consciousness; Neurosurgery; Brain Stimulation, Psychiatric and neurodegenerative diseases and disorders*), the second group of topics is associated with the ethics of technology-based interventions on cognition (*Addiction, Psychopharmacology, Enhancement*) and the third group comprising topics associated with the neuroscience of ethics (*Moral Theory, Philosophy of Mind, Neuroimaging*). We also found that *Neuroimaging* is one of the central subject-categories that besides forming a part of the third group is connected to almost all of the other subject-categories (discounted *i*_*a, b*_, above 0.7 for all connections to other subject-categories, cf. Figure 4). The subject-categories of *Neuroscience and society* and *Legal Studies* form a central part in the network of topics by displaying connections to most of the other subject-categories.

### Neuroethics publications in the social sciences and humanities and in the biomedical sciences

Having identified neuroethics as thematically being an endeavor that falls between very diverse and partly overlapping research fields, we wondered how the rise of neuroethics in the last quarter century maps on the landscape of scientific journals publishing contributions in the field. Scientific journals contribute to delineating and structuring scientific disciplines, through their differentiated editorial selections, or more mundanely through the central role they are made to play in the decisions for career promotions for researchers. For this reason, we found it particularly interesting to determine where contributions in neuroethics are published. Hence, using our expertise from our previous research in neuroscience and neuroethics, we distinguished journals of two broad categories: journals focusing on empirical research in medicine and natural sciences were labeled as “biomedical science journals,” while journals publishing primarily qualitative research in the social sciences, philosophical enquiries or ethical issues, were labeled as “social sciences and humanities journals” (SSH journals for short) We controlled this grouping of journals by drawing on the ISI journal classification scheme (cf. Supplement 2 in the Supplementary Material). First, we listed the journals per categories and the number of neuroethics articles per journals per category. For the analysis, in each category all journals from the MND containing three or more neuroethics publications from the period 1995–2012 were taken into consideration (i.e., those with the highest number of publications per subject-category). Finally, in each subject-category, the number of SSH journals and biomedical sciences journals among those most relevant journals per subject-category was determined and the number of articles was counted. The results are displayed in Table 1.

As the table shows, the overall size of the subject-category in terms of the total number of articles correlates with the total number of scientific journals within the subject-category. Hence, *Psychiatric and neurodegenerative diseases and disorders* is also the largest subject-category in terms of the number of relevant scientific journals. This holds for the whole scale of subject-categories: the larger the category in terms of articles, the larger it is in terms of the number of relevant journals. A further general finding is that there are overall many more biomedical science journals that have published articles relevant for neuroethics, than there are SSH journals that have published work relevant for neuroethics. The ratio in our dataset is 2.5:1. Except for four subject-categories (*Philosophy of Mind and Consciousness, Legal Studies, Neuroscience and Society* and *Enhancement*) the ratio of biomedical science journals to SSH journals is greater than one, indicating that the bulk of neuroethics research since 1995 has been published in journals that usually do not conceive of moral, legal and social questions of biomedical research as their proper topics. This general dominance of biomedical science journals in the emerging field of neuroethics also reflects the scientific background of neuroethics researchers (see next section).

The four subject-categories with a biomedical science journal to SHH journal ratio lower than one, however, do not come as a surprise: *Philosophy of mind* and *Legal Studies*, even though these are fields, which in some parts strongly refer to neuroscience research, are very established disciplines of the Humanities and Social Sciences, which have their own traditional and highly recognized corpus of relevant journals. Taking into account the general trend toward an approach to theory formation informed by neuroscience, it is not surprising that neuroethics research also encroaches upon these established SSH-Journals. Also for the category *Neuroscience and Society*, a surplus of publications in SSH Journals is plausible, because the category's content focusses strongly on the influence of neuroscience in the public. This reflective perspective on neuroscience is typical for anthropologists and sociologists or historians of science and hence has its genuine place in SSH journals. More surprising, however, is the ratio of biomedical science journals vs. SSH journals for the subject-category of *Enhancement*, particularly if one regards the strong ties of this research field with *Psychopharmacology* and *Addiction* research. In the Section The Institutionalization of Neuroethics we will explain this finding and the similarly unexpected distributions of papers on the two groups of journals in the subject-categories *Moral Theory, Brain Death/Severe Disorders of Consciousness*, and *Neurosurgery* with regard to the historical development of neuroethics.

### Influence of the journals “neuroethics” and “AJOB neuroscience” on publication patterns in neuroethics

This analysis only allowed a glimpse into the larger picture of publications in neuroethics, because we did not identify changes in the publication patterns of different subject categories over time. Nonetheless, neuroethics has also begun to establish itself as a discipline by the foundation of two very central scientific journals, Neuroethics (founded in 2008) and the American Journal of Bioethics: Neurosciences (AJOB Neuroscience for short) (founded in 2010). As the foundation of scientific journals especially dedicated to a certain research field can be expected to change publication patterns within the field, we wondered what impact these two widely acknowledged journals had on the distribution of neuroethics publications in scientific journals. To estimate this influence we compared the distribution of papers within the different subject categories in the interval between 2004 and 2008 and the interval between 2008 and 2012 (cf. Table 2).

**Table 2:** **Influence of the emergence of Neuroethics and AJOB Neuroscience on the publication patterns in neuroethics**.

	**Number/total number of journals**	**Total number of publications**	**Number of publications in Neuroethics and AJOB-N**
1995–2007	49/105	704	0
2008–2012	86/105	1592	374

**Figure 5:**
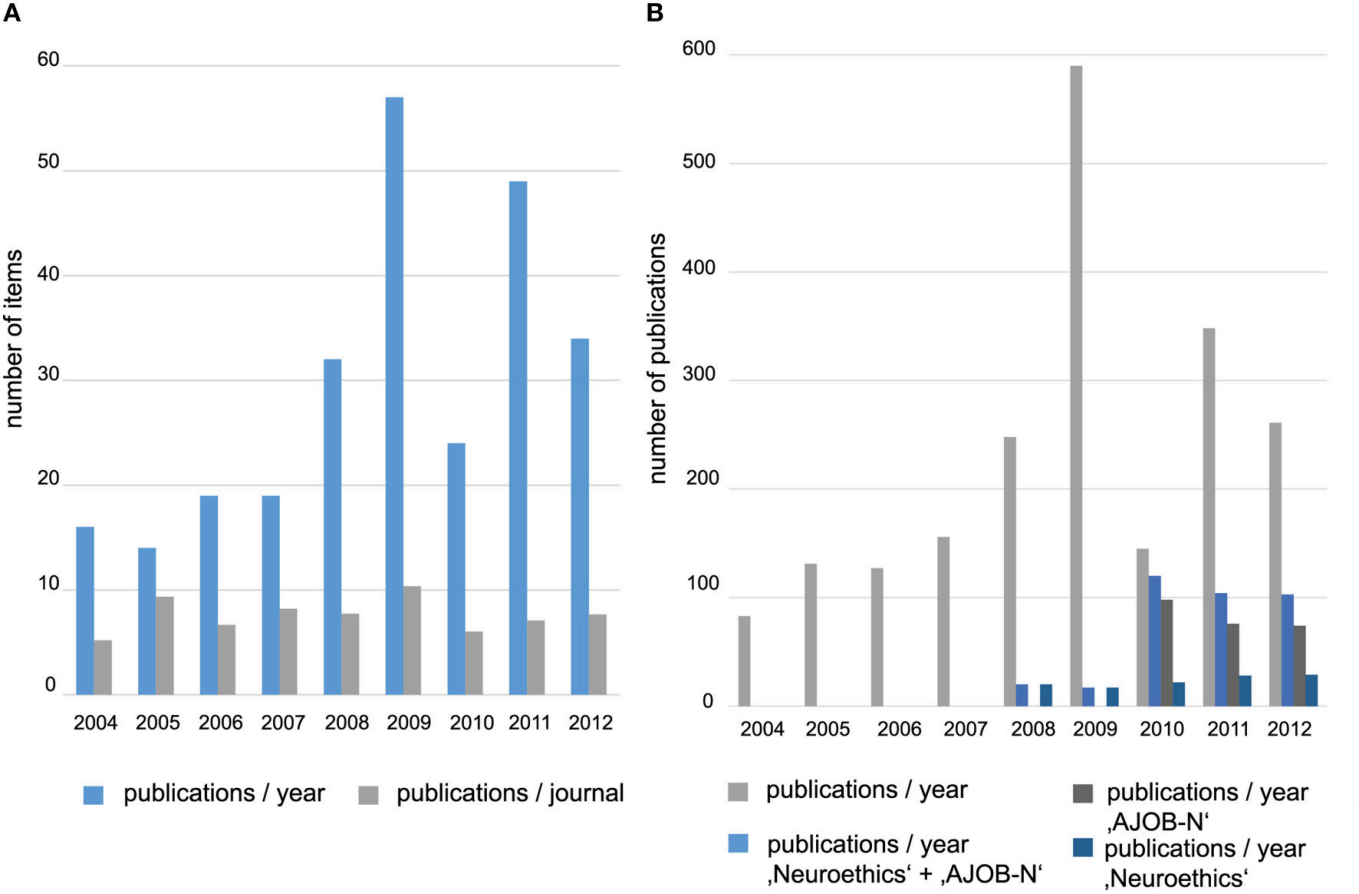
**Influence of the journals Neuroethics and AJOB Neuroscience on publication pattern in neuroethics. (A)** The diagram displays the increasing number of publications in neuroethics and the increasing number of journals publishing articles belonging to the field between 2004 and 2012. While the number of publications increased, the ratio of publications per journal remained relatively constant over the years. **(B)** Diagram shows the influence of the two journals Neuroethics and AJOB-N for the development of neuroethics as a field. After the first appearance of these journals in 2008 and 2010 respectively, there can be observed a slight decrease in the overall number of publications. In this time interval, a share of 29.9–82.6% of all publications appeared only in Neuroethics and AJOB-N. Between 2008 and 2012 on average 32.6% of all neuroethics publications were published in these two journals.

Our analysis indicates that the foundation of the two journals significantly contributed to the structuring of the field, but that nevertheless, less than a quarter of all relevant papers are published in these journals (cf. Figure 5). In the period between 2008 and 2012, only about 374 contributions were published in these two dedicated journals (120 in *Neuroethics* and 254 in *AJOB Neuroscience*). Based on the corpus of the MND, this makes up about 23.4% of all publications relevant to neuroethics in this period.

### The institutionalization of neuroethics research

**Figure 6:**
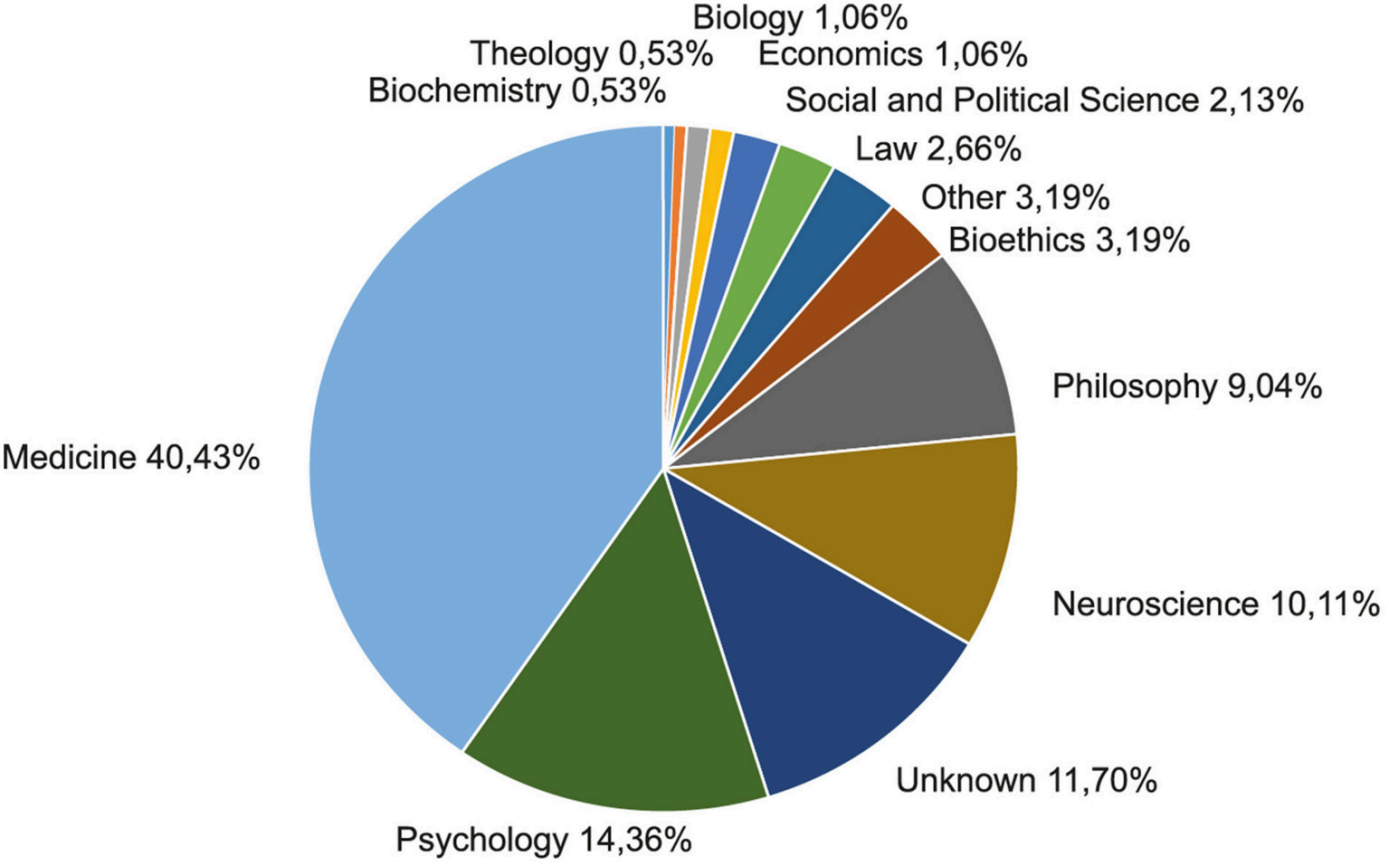
**Academic education of relevant researchers in neuroethics**. The chart visualizes the size of different groups of researchers engaging in neuroethics as represented by their discipline of initial academic education. To describe the distribution of the academic background of neuroethics researchers we used the 20 researchers with the highest publication output in each of the subject-categories as a measure.

Besides the study of disciplinary journals, the institutionalization process of a research field can be described by the development of research centers and research groups that focus on questions relevant in the described subject-categories. In order to further survey the institutionalization of neuroethics we therefore examined the current affiliation of the most relevant researchers of each subject-category as well as their educatory background. As a first step, we determined the most relevant researchers in each subject-category by retrieving the names of the 20 researchers with the highest number of publications from each of the subject-categories. When two or more researchers with the same number of publications were ranked as number 20 in the respective subject-category, we preferred those with the higher number of first author publications over those with second or third author publications. As several researchers belonged to the top 20 in more than one subject-category the procedure provided a list of the names of 198 researchers. Queries in Google, WoS, and in the MND allowed us to add the academic discipline(s) in which these researchers were initially trained, their highest academic degree obtained, their current affiliation and the country of their current host institution (cf. Supplement 3 in the Supplementary Material). Linking these data revealed that a minority of the top researchers in neuroethics have a dual academic degree or have been educated in an interdisciplinary academic research program from the start (16.2%). Hence, more than two thirds of the neuroethics researchers have been trained in only one discipline (68.7%). The data we obtained from our research showed a strong variability in academic backgrounds of relevant neuroethics researchers (see Figure 6).

General findings indicate that a majority of researchers in the field of neuroethics have a background in medicine (40.43%), while another 24.47% have entered Neuroethics after having been trained in psychology (14.36%, including behavioral science, cognitive psychology, social psychology, experimental psychology, cognitive science) or neuroscience (10.11%, including psychobiology, neurological science). These finding support the observation that neuroethics is primarily fueled by research from the empirical medical sciences and the life sciences (including psychology). Scientists with a genuine competence for normative and conceptual questions, such as scholars of philosophy and bioethics seem to be not adequately represented in neuroethics, at least if the normative character of many research questions and debates in the field is considered. Nonetheless, according to our database scholars with an initial or additional training in philosophy or bioethics form a proportion of about 17.02% of the authors in neuroethics.

**Figure 7:**
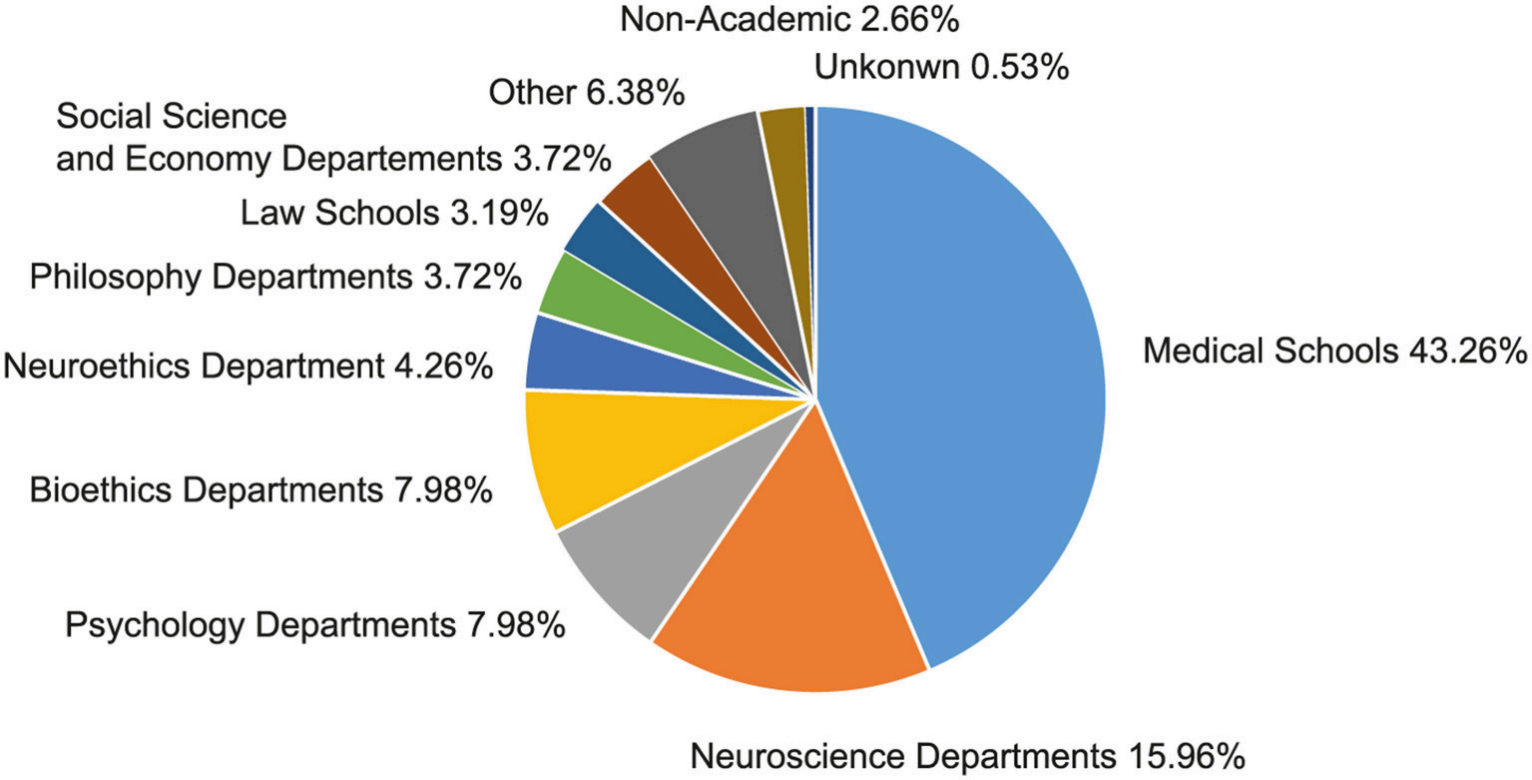
**Departmental affiliation of relevant neuroethics researchers**. The chart shows the size of different groups of neuroethics researchers as represented by their current academic affiliation (as of autumn 2014). To describe the distribution of the academic background of neuroethics researchers we refer to the 20 researchers with the highest publication output in each of the subject-categories as a measure.

With regard to disciplinary affiliations neuroethics, researchers display surprisingly little mobility. Even though neuroethics is a highly interdisciplinary field and even though there aren't yet any structured programs for becoming a “neuroethicists” so that one might consider a high disciplinary mobility to be an advantage on the job market, we found that many of the scientists who publish in neuroethics stay affiliated with departments of the discipline in which they were initially educated. Most researchers still have their affiliation with a medical center or a science research institute (67.2%). While most of the identified neuroethics researchers are affiliated with university medical centers (43.26%), another 15.96% work in neuroscience laboratories and another 7.98% in psychology departments at universities or private research centers. Hence, also with regard to affiliation, the social sciences and humanities are underrepresented in neuroethics. Only 7.98% of the researchers in our dataset were affiliated with a bioethics department, and another 4.26% with a department specifically dedicated to neuroethics. The complete spectrum of the social sciences (sociology, political sciences, economics) only made up 3.27% of the departments hosting neuroethics researchers with a high publication output (cf. Figure 7).

Besides the US, researchers in neuroethics with a high publication output primarily work in Canada, the UK, Germany and Australia (cf. Table 3). All these industrial nations of the western hemisphere have also been at the forefront in neuroscience and medical research. Hence, from a wider perspective of a technology-driven understanding of neuroethics this finding was expected. This result follows a trend that has been reported by Lombera and Illes (2009), who equally found that neuroethics research primarily centers in high-income countries as defined by the World Bank. However, in comparison to their investigation into the international dimensions of neuroscience from a quantitative perspective on the published research output, more contributions from countries like France, the Netherlands, Israel or Switzerland would have been expected. However, as we did not count for the hosting countries of authors from all publications in our database, the international dimension of neuroethics might be broader than our list suggests.

**Table 3:** **Countries hosting neuroethics researchers with high publication output according to MND**.

**State of host institution**	**States' share of world-population (%)**	**Number of high-output neuroethics researchers**
Australia	0.329	11
Belgium	0.15	3
Brazil	2.82	3
Canada	0.49	19
Denmark	0.078	1
Finland	0.075	2
France	0.88	1
Germany	1.12	15
Italy	0.83	3
Japan	1.74	5
New Zealand	0.0683	2
Singapore	0.076	1
South Africa	0.75	2
Sweden	0.13	1
Switzerland	0.11	4
Thailand	0.89	1
The Netherlands	0.233	3
Trinidad and Tobago	0.018	1
United Kingdom	0.89	18
United States of America	4.42	91
Unknown	–	11
Total		198

## Discussion

We started our investigation by discussing different approaches to define neuroethics. To shed new light on these stipulative top-down approaches we aimed at complementing this discussion by an in-depth analysis of the recent development and the current landscape of neuroethics literature. Our empirical, bottom-up analysis revealed not only a constant quantitative increase in the neuroethics literature over the years but also a constant relative size of the different subject categories. Hence, our study confirmed for neuroethics as a whole what has been reported by previous studies with a more limited focus (Seixas and Ayres Basto, 2008; Lombera and Illes, 2009; Garnett et al., 2011; Gooray and Ferguson, 2013). It also shows that most issues now discussed under the label of Neuroethics have indeed gained much more attention since the middle of the 2000s than they have gained in the antecedent decade and that the issues as such are not new and have not changed much over the years. However, some topics in neuroethics especially those associated with ethical questions of clinical research (*Brain Death, Medical Research*) and technological developments (*Enhancement, Neuroimaging*) in neuroscience, have profited several years earlier from this gain of attention than topics related to other branches of neuroethics (*Moral Theory, Brain Stimulation*). A small exception to this trend is the ethical debate about issues in *Deep Brain Stimulation* that according to our data developed its strongest increase only after the year 2008, whereas it was much less discussed in the years before. Because the technology was in use for the treatment of Parkinson's disease at least since the 1990s, the development of this subject-category reflects the extension of possible applications of DBS onto psychiatric diseases.

The increase of publications under the label of neuroethics correlates with a general increase of publications in neuroscience (Christen, 2010; Matusall et al., 2011) and could be interpreted as a consequence of the Decade of the Brain and the subsequent intentions to enhance awareness of the ethical, social and legal implications of neuroscience. Our findings further indicate that the bulk of neuroethics research during the last 25 years has been concerned with issues that are primarily of interest when neuroethics is construed from health-care or technology-driven perspectives (Racine, 2010).

Because publications about the neuroscience of decision-making in moral contexts (i.e., the subject-categories of *Moral Theory* and *Philosophy of Mind and Consciousness*) are generally smaller in number and have not increased their share of the neuroethics literature in the recent years, the mainstream of neuroethics research is tackling with ethical questions within the established theoretical frameworks of bioethics. This finding is consistent with those of Lombera and Illes (2009), who showed a dominance of publications on the “ethics and practice of brain science.” However, it does not mirror observations of Gooray and Fergusson (Gooray and Ferguson, 2013), who rank literature on moral philosophy and moral psychology as the second most abundant issue in neuroethics after issues in cognitive enhancement. This last opposing result, however, is very likely due to the use of different data-sets. Goory and Ferguson distinguished in their analysis between journal articles and books (which we did not) and restricted their search for neuroethics literature to a list of only 12 preselected journals. Our dataset instead comprised contributions from 105 different sources. The list of journals used by Gooray and Fergusson is extremely restricted insofar as it did not include any journal that is specifically concerned with clinical and health care issues in neurology and psychiatry and did not include any journal on applied ethics or bioethics except AJOB-N and Neuroethics.

Our corpus of literature instead developed as an open access multimodal compilation of neuroethics literature and from the beginning, and did not restrict possible entries according to the criterion of appearance in one of a restricted number of journals (see Method Section). Because of this, we consider our description of the field more accurate and more reliable. Our findings, however, do not contradict those of Christen (2010), who reports a proportional increase in papers concerned with “moral neuroscience” compared to the general increase of papers in “neuroscience.” This seeming divergence is, however, only due to the diverging comparative measure. Christen observed the increase in comparison to publications in neuroscience in general, while we point out that the share of papers concerned with “moral neuroscience” does not vary over time, if compared to papers concerned with “neuroethics” in general. It is, hence, plausible to notice a general increase in neuroethics literature after 2002 in relation to literature in neuroscience in general.

A more in-depth quantitative analysis further revealed that some topics in neuroethics that are often considered to be central to the field, are indeed not as important. Even though in general the number of publications in the ethics of neuroscience branch of neuroethics has predominated the number of publications of the neuroscience of moral decision-making branch, within the first, issues such as cognitive enhancement or deep brain stimulation have shown to be quantitatively much less important than questions of clinical ethics with relation to psychiatric and neurodegenerative diseases. Much more than being predominantly a technology-driven research field, in the last 25 years neuroethics has been mainly driven by questions of medical and health-care ethics. Hence, contrary to the view communicated for example in the study of Gooray and Ferguson (2013) our data not only indicates that *Enhancement* is actually one of the smallest subject-categories of neuroethics, but also that it has been an issue even before 2002. What is more, our data also shows that the subject-category *Enhancement* did not increase in a greater proportion compared to other subject-categories after 2002. This finding is surprising, considering the fact that those, who maintained the MND since 2008 were primarily engaged with research on cognitive enhancement and were hence particularly aware of the literature in this specific subfield of neuroethics. This entanglement of the researchers with cognitive enhancement obviously did not lead to an overrepresentation of enhancement literature in the MND.

Even though only a middle-sized subject-category, our data shows that *Neuroimaging* should be seen as one of the most important issue in neuroethics. This is because this subject category is linked to most of the other subject categories in neuroethics and has a potential to bridge the gap between different branches of the discipline. This significance of neuroimaging for some parts of neuroethics is in line with the purported importance of imaging methods for neuroscience in general. However, it also indicates that particularly research in neuroeconomics and in the neuroscience of ethics has an affinity with this method. Similarly the strong connection of *Neuroimaging* with *Legal Studies* could be seen as mirroring an ongoing debate by law scholars and neuroscientists about the ethical and legal problems of mind-reading and incidental findings during neuroimaging procedures. This finding is in line with work by Garnett et al. (2011), who found that from a quantitative point of view, there is only a limited number of articles relating to neuroimaging via fMRI that also treat ethical issues of the use of this technology. Other quantitatively important branches of neuroethics are represented by those subject-categories concerned with issues closely related to medical ethics, specifically those concerned with ethical issues in psychiatry. Considering the broad basis of the MND this is not surprising, because questions of patient autonomy and patient ability to give informed consent have been particularly important in psychiatry even before the rise of neuroethics as a distinct research field (Bloch and Green, 2009). On the other hand, the ongoing turn toward neurological explanations of psychiatric illness has shed new light on these issues making them an obvious topic for neuroethics. Taken together, our analysis of the quantitative development of neuroethics publications underscores conceptualizations of neuroethics that emphasize the roots of the discipline in the area of health-care and medical ethics.

### The relation between different branches of neuroethics

Our analysis of the relation between different research topics addressed in neuroethics supports the view of neuroethics as a multifaceted endeavor in which different research foci do not necessarily interconnect. We found three tightly interconnected groups of thematically strongly interrelated subject-categories that can be seen as mapping the three perspectives on neuroethics suggested by Racine (cf. Figure 4). The first group displays particularly strong connections between the subject-category *Brain stimulation* with the subject-categories *Neurosurgery* and *Psychiatric and neurodegenerative diseases and disorders*. These connections are easily explained by the fact that invasive forms of brain stimulation used in the treatment of neurodegenerative disorders such as Parkinson's disease and various other diseases require surgical intervention. The second group is characterized by the strong ties between the subject-categories *Enhancement, Psychopharmacology* and *Addiction* and indicates that these issues are frequently discussed in relation with each other. This result is not very surprising considering the fact that in neuroethics, the discussion on cognitive enhancement has originally been driven by the phenomenon of off-label use of prescription drugs such as Ritalin and Prozac (Kramer, 1993; DeGrazia, 2005; Elliott, 2011). This phenomenon itself, however, could be described as well as a phenomenon of drug abuse, which would implicitly relate it to the topic of addiction (Outram, 2012). The result is also very interesting in light of our finding that Enhancement is quantitatively a relatively small branch of the neuroethics literature, because it suggests that the publications tagged in the thematically related subject-categories of *Psychopharmacology* and *Addiction* do not simply add up with Enhancement to form one larger subject-category. Finally the third group comprising subject-categories including empirical and theoretical issues associated with the neuroscience of ethics branch of neuroethics, is very well connected to the subject-category of *Neuroimaging*. The subject-category *Moral Theory*, which forms part of this cluster has, for example, its strongest ties with *Social and economic neuroscience, Philosophy of mind and consciousness* and *Neuroimaging*. Even though this subject-category is quantitatively speaking rather large, it is only weakly connected to subject-categories outside the group.

Particularly interesting with regard to the distinctions drawn by Racine, is our observation that the more theoretical approaches to neuroethics (i.e., neuroscience of decision-making in moral contexts) that represent the knowledge-driven approaches and that are grouped in the subject-categories of *Moral Theory* and *Philosophy of mind and consciousness* hardly connect with subject-categories relevant for health-care and technology-driven approaches to neuroethics. Even the connections we found with the subject-categories *Neuroimaging* and *Brain Death / Severe Disorders of Consciousness* rather point to the plausible fact that new theoretical approaches in moral theory and in the philosophy of mind are well informed by neuroscience research and by case studies that reveal knowledge about a wide range of consciousness phenomena. However, at the moment this empirical knowledge does not yet interrelate with the topics of practical neuroethics. This suggests that there is a significant divide between the two branches of neuroethics distinguished by Roskies and that knowledge-driven approaches to neuroethics are currently not dominant in the field. Hence, it is likely that only a minority of researchers in neuroethics actually shares the idea of an informative or even constitutive role of neuroscience for ethical theory. The gap between the two branches of neuroethics is also reflected by publication patterns, because the thematic distribution of neuroethics publications in biomedical science journals and SSH journals can be interpreted as mirroring the divide between neuroscience of decision making in moral contexts and applied neuroethics, i.e., ethics of (clinical) neuroscience. While publications in the applied branch to a large extent appear in biomedical science journals, the theoretical discussions tackling questions about the relevance of neuroscience research into the foundations of moral behavior are primarily discussed in SSH journals. In spite of this observation, however, the divide between the two branches of neuroethics should not be overstated. Our analysis is not fine-grained enough to elucidate the divide on the level of single publications. So far we have only shown that a cleavage between the two subfields of neuroethics is mirrored by a tendency to publish more theoretical and foundational work that is inspired by empirical findings from neuroscience with SSH journals. And it should be kept in mind that the two journals explicitly dedicated to neuroethics (*Neuroethics* and *AJOB-N*), which were started only in 2008 and 2010 respectively and which we grouped both in the SSH journal category explicitly focus on issues that relate to both branches, and thereby work on overcoming the gap.

Whether this gap will be overcome in the future and whether neuroethics will follow the dynamics pointing at more knowledge-driven approaches to the field, cannot be inferred from our data. What we see, however, is that the disciplinary background of current neuroethics researchers speaks in favor of the hypothesis that many trained empirical researchers engage with normative questions of applied ethics. A large amount of publications in neuroethics are produced by researchers with an academic background in neuroscience, psychology and medicine. In comparison, researchers with a background in philosophy, bioethics and the social sciences are marginal in the field. This finding fits with the data communicated by Gooray and Ferguson (2013), who also report a dominance of neuroscientists and physicians, even though they found a higher proportion of philosophers in the field than we did. Does this mean that neuroscientists and physicians, who do empirical work, start to address questions that have traditionally been a subject for philosophers and social scientists? It is not obvious how to interpret this result. One possibility is, to see neuroethics within a framework of an ethics in the sciences, that is primarily driven by the reflection of ethical issues scientists or medical doctors confront during their everyday work. This shows not only the need for an ethical approach to the practice of neuroscience and the drive to discussion of ethical issues that comes from the sciences themselves, but also the need for participation and active involvement of empirical scientists in the ethical discourse.

Another interpretation, however, would not be as flattering for neuroethics. It suggests that our findings indicate that a large part of neuroethics is the result of the engagement of neuroscientists, psychologists and medical doctors with normative and conceptual issues, for which they have not sufficiently trained in their academic education. To better assess the plausibility of these hypotheses, it would be necessary to consider the contents of the publications. For one might object that many of the publications written by neuroscientists are not actually on the ethics of neuroscience but about the neuroscience of decision-making and reasoning in moral contexts. Given the number of neuroscientists in the field and the amount of papers in the database that deal with the neuroscience of decision-making and moral reasoning, this is, however, extremely unlikely. Only about 8% of all articles relate to questions of moral theory and neuroscience (i.e., the subject-category moral theory), whereas about 56% of all top researchers in neuroethics have a background in neuroscience, psychology and medicine. Hence, it is much more plausible to assume that many neuroscientists have actively contributed to research on the ethical implications of neurotechnology and neuroscience research. At least to a certain degree this implies that the ethics of neuroscience is profoundly shaped by the knowledge and research agendas of neuroscientists themselves. This need not necessarily be regarded as a problematic dilettantism of the neuroethics community, but may be an acknowledgment of the fact that ethical assessments of issues associated with neurotechnologies and clinical neurology structurally rely on knowledge of specialists in the field. However, seen from a more sociological angle, this finding also indicates that the accusation of a lack of distance between neuroscience and neuroethics cannot be easily rejected. This lack of distance, expressed by an uncritical fascination of neuroethicists with the promises of (future) neuroscience and -technology, might indeed become problematic, if the research agendas of neuroscientists started to define the extent of legitimate ethical objections (De Vries, 2007; Brosnan, 2011).

The critique of neuroethics that flows from such a purported lack of distance between neuroscience and neuroethics has two main points of reference. The first is the worry that neuroethics, instead of critically assessing and regulating the research and practice of neuroscience and neuro-medicine, might engage in generating unrealistic and overoptimistic expectations about the future of neuroscience research (Quednow, 2010). This not only has the side-effect to effectively generate further needs for Neuroethics research, but also to waste limited resources to engage with unrealistic and improbable ethical scenarios (Brosnan, 2011; Hoyer and Slaby, 2014). The second critique sees a disadvantage in a tight entanglement of neuroscience and ethics, because neuroethicists, who are fascinated by the technological developments in neuroscience but at the same time not aware of the history and structure of bioethics, might tend to squander resources on questions that have already been treated in the context of the assessment of other biotechnologies (Parens and Johnston, 2007). Instead of reinventing the wheel of bioethics, neuroethics should be aware of the convergence of many ethical questions deriving from context of technologies as diverse as genetic testing, nano-medicine, and fMRI-studies. Together, these criticisms have yielded the accusation that the development of neuroethics is at least partly a byproduct of a hype of the neurosciences in the recent decades (Choudhury and Slaby, 2012).

### The institutionalization of neuroethics

Besides the development and current structure of the thematic landscape of neuroethics, we were also interested in the institutionalization of the field. Institutionalization is an important point of view to assess the demarcation of a research field from other areas of investigation. Besides defining a discipline by its specific topics and research questions an investigation into its research infrastructure provides additional information. Some important indicators suggests that neuroethics is currently on the way to establish itself as a discipline distinct from bioethics and from the neurosciences respectively. This is mirrored by the fact that a scientific society for the discipline has been founded in 2006 (the Neuroethics Society), which in 2011 aligned with other disciplinary groups and networks (International Neuroethics Network) to form the International Neuroethics Society. It is further reflected by the foundation of two journals (*Neuroethics, AJOB-N*) with the explicit aim of providing a platform for neuroethics research. Our analysis indicates that these journals significantly contribute to discussions in the field but that nevertheless only about a quarter of all relevant papers in the last quarter century has been published in these journals. In the period between 2008 and 2012 only about 374 contributions were published in these two journals. Based on the corpus of the MND, this makes up about 23.4% of all publications relevant to neuroethics in this period. This is of course still a very high amount considering the fact that both journals together comprise only 2.1% of the journals and book chapters that contributed to the MND. Even though this finding underlines the great importance of these two journals for the neuroethics community, our data indicate that the dominance of these journals should not be overstated. Hence, the data reported by Gooray and Ferguson (2013), which shows a share of publications in *Neuroethics* and *AJOB-N* oscillating between 78 and 95% between 2008 and 2012, should be put into perspective. Instead, our analysis shows, that despite the fact that neuroethics has established its own publication organs and despite the fact that these publication organs obviously play a leading role for the development of the field, work on issues in the field is still today widely spread on a large variety of scientific journals and books.

It was particularly striking that subject-categories could be distinguished according to the biomedical science journal to SSH journal ratio of the published articles. Besides the subject-category *Enhancement* that displayed a ratio of biomedical science journal to SSH journals greater than one, we found similar rations for four other subject-categories. In the following we will comment on three of them. In the case of *Enhancement* this ratio can be explained by the historical observation that the neuroethical enhancement-debate originated in other branches of bioethics (i.e., gene-ethics, ethics of human reproduction) (Harris, 2009; Savulescu et al., 2011). These roots might explain the dominance of SSH journals in this subject-category. For *Moral Theory*, instead, this finding could partly be explained by the literature emerging from neuroscience research into the brain mechanisms involved in decision-making processes in moral contexts. This descriptive strand of research has profited much from neuroimaging technology in the last quarter century (Greene et al., 2001, 2004; Moll et al., 2002a,b; Kahane et al., 2012) and, hence, is adequately placed in journals from the biomedical sciences. The impression of a shift toward the medical and natural sciences in the field of *Moral Theory* is, however, diminished by the observation, that the majority of articles in this subject-category still get published in SSH journals. This hints to a trend in many biomedical science journals to superficially take up issues form a neuroscience perspective, which relate to moral theory and ethics and that are traditionally discussed in a subset of philosophical SSH-journals. This leaves the bulk of the discussion to a small number of SSH journals. Hence, it seems that issues in moral theory are still predominantly discussed in SSH journals, but are increasingly taken up as an issue of interest in many biomedical science journals in the course of theoretical efforts to draw a naturalistic picture of the phenomenon of morality (Casebeer, 2003; Casebeer and Churchland, 2003; Greene, 2003). We found a comparable pattern for the subject-category *Brain Death/Severe Disorders of Consciousness*. While the positive biomedical science journal to SSH journal ratio hints to issues discussed from a biomedical science point of view, it seems that the majority of the discussion take place in a few SSH-Journals. This could be due to the discussion on the definition of brain death and its role in the normative issue of declaring a person dead. This classical question in medical ethics cannot be addressed without knowledge from the biomedical sciences, but as it also has a severe normative impact the discussion of this topic in SSH journals and particularly in bioethics journals is justified.

Despite the growing organization of neuroethics by scientific journals and research organization, the need for neuroethics research has not yet translated into a corresponding global research infrastructure. Our data indicate that neuroethics was formed primarily inside the established institutional structures of the biomedical sciences and bioethics and only to a much smaller extent in the traditional humanities and the social sciences. Regarding the fact that we found no significant mobility of researchers trained in one discipline toward positions in departments distinct from that discipline (except six philosophers, who worked for bioethics or neuroethics institutes instead of philosophy departments) researchers of neuroethics seem to be as conservative as the academic system with its marked-off disciplines with regard to their affiliations. Eventually the ongoing establishment of interdisciplinary research centers dedicated specifically to neuroethics—especially in the USA and Canada - might shift this disciplinary bias in the future toward a more active involvement of the social sciences and the humanities. This hypothesis is underpinned by our finding that researchers affiliated with research centers and departments of neuroethics mainly work in the US and Canada. However, our data also revealed that only a very small number of the neuroethics researchers with high publication output are currently affiliated with a special neuroethics research center (NRC). This may of course be due to the fact that most of the NRCs are interdisciplinary institutions themselves, so that many researchers do not have their primary affiliation with an NRC but with another institution (i.e., Neuroscience Laboratory, Clinic or Philosophy Department). This finding is supported by the fact that in our data-set there were three NRCs and two larger Research Units that hosted at least one of the top neuroethics researchers (*Canadian National Core for Neuroethics, University of British Columbia; Center for Neuroscience and Society, University of Pennsylvania; Oxford Center for Neuroethics, University of Oxford; Neuroethics Research Unit, IRCM Montréal* and the *Mind, Brain Imaging and Neuroethics Research Unit, University of Ottawa*). This and the observation that many other research groups concerned with neuroethics are part of (bio-)ethics departments, speaks in favor of the claim that neuroethics research is not as independent from research in bioethics on the one hand the neurosciences on the other as neuroethicists would like to see it.

### Implications for the future of neuroethics

In the introductory section, we emphasized that every account of neuroethics is partly stipulative and partly descriptive. By using bibliometrical data, we have investigated the structure of the phenomena any account of neuroethics should refer to. This descriptive approach to neuroethics could not decide whether a technology-driven, a health care-driven or a knowledge-driven approach to neuroethics is most adequate. However, by providing an empirical description of the development of neuroethics research in the past quarter century and about the mutual interrelations of topics sailing under the flag of neuroethics, it revealed the diversity and vast extent of the research field. On the other hand, any stipulative top-down approach to neuroethics can neither help drawing boundaries within the research field nor help to declare some issues as more central to neuroethics than others. Certain evaluations and interests, hence, guide any conceptualization of neuroethics. With the diversity of research topics in neuroethics in mind, we showed that many of the current conceptualizations actually relate to what in the scientific literature is understood as neuroethics. Nonetheless, there are conceptualizations of neuroethics that are more inclusive than others. Hence, from our empirical point of view, a concept of neuroethics with a single focus on the neuroscience of decision-making processes in moral contexts would be overly narrow, because quantitatively speaking the neuroethics literature on this area of research is comparatively small. But also a concept of neuroethics, which in the best tradition of a technically induced bioethics would focus mainly on the ethical, legal and social consequences of neurotechnological innovations, would still draw the boundaries of the field much too tight. Even though the quantity of neuroethics research addressing the conditions and consequences of technology implementation is much larger than that of the literature of neuroscience of moral decision-making, it would be somewhat arbitrary to ignore the close connections between the ethical implications of the use of neurotechnologies and questions of clinical neuroethics. Many technical innovations in neuroscience do not only have a direct impact in research contexts but also in clinical settings and in everyday life. Furthermore, such technologies are often developed as medical devices and, hence, it would be difficult to completely ignore this context, when technology assessment is at stake.

Drawing on our data about the temporal development of different research topics, we must conclude that none of the mentioned approaches to neuroethics has yet become dominant. Instead they are not mutually exclusive and sometimes even complement each other. It is in fact true that our data analysis revealed a significant gap between empirical work in neuroethics that addresses the neural basis of decision-making in morally relevant contexts and the questions addressed by applied neuroethics. However, it remains an open question whether neuroethics should work on overcoming this gap and if this is possible at all. It has been argued for both alternatives in the past, and it is likely that this dispute will continue, because our analysis does not underpin the hypothesis that neuroethics will enter a state of transition in the coming years. Rather neuroethics currently is a notably stable field of research that has not significantly changed in the number of publications per year since 2009 and which displays a very robust continuity of research questions and relevant topics. The fact that almost all topics discussed within neuroethics today have been present in debates in medical ethics, pharmacology or philosophy of mind shows this particularly clear. Currently neuroethics as a discipline amounts to bringing together these primarily distinct topics under one label, but not necessarily under one unified research agenda.

From this point of view, it is difficult to assess the potential of neuroethics to establish itself as a discipline of its own distinct form the neighboring research fields in neuroscience, bioethics and philosophy of mind. Even though neuroethics has taken some important steps in this direction on the institutional level (scientific journals, scientific communities, a few research centers), the broad distribution of neuroethics research over a very large corpus of journals and the rather homogenous academic background of successful neuroethics investigators in biomedicine and neuroscience speak against this hypothesis. Suiting this our data indicate that the entanglement of neuroethics with neuroscience is still rather strong. Whether neuroethics will become more independent in the future should be monitored by further studies addressing literature from a much more recent timespan. A regular monitoring of the field would not only help to understand its development but also to gain adequate and actual descriptions of the field that are sufficiently informed by the factual phenomenon of ongoing neuroethics research.

### Limitations

It could be argued that our study is limited in two important regards. First, the quality of the database limits the validity of our results, and second, one might object that our analysis of topics and networks in neuroethics crucially depends on the reliability of our system of subject-categories.

We believe, we can meet the first objection with reference to the fact that neuroethicists themselves know best, what their research is about. The MND was built as a resource for neuro-ethcists by neuroethicists. Hence, the versatile collection of material making up the MND should be more representative of neuroethics than the content of other, alternative databases. Second, this open, participatory structure prohibited that the regular scans of new publications turn out all too subjective.

Concerning the second objection, we are well aware that the differing number of defining keywords per subject-category might raise doubts about the system's reliability. Prima facie the probability of a paper to be tagged in a subject-category defined by more than 20 key-words can be expected to be much higher than the probability of a paper to be tagged in a subject-category defined by much fewer keywords. Hence, the differing length of keyword lists in our approach might undermine the reliability of the applied subject-categories. This objection, however, can be overruled by the observation that imposing a balance in the lists of keywords leads to a less specific matching of papers and subject-categories than in the lists we used. Choosing and approach allowing (a) for a constant number of 16 keywords per subject-category, (b) for tagging of a paper only when at least two terms within their headlines, abstracts and keywords matched with the keywords defining a category, and (c) for automatic labeling of papers to belong to a category only, if terms corresponding to the topic's label were present in the text, produces a much less reliable categorization. This alternative procedure not only leads to an increase in papers categorized in topics that were defined by more keywords than in the original procedure (for example the category “Addiction,” which was defined by nine keywords and relates to 55 paper in our approach, relates to 92 papers in the alternative approach, for further details c.f. Supplement 4 in the Supplementary Material), but also to a large overall decrease in the number of topics attributed to papers. Using this alternative procedure a quarter of all topics could not be detected because the constraints (a)–(c) left some informative language features unused, which were located by our approach. We find that it is preferable to keep the procedure, which has the best recall.

## Author contributions

JL and CL developed the system of subject-categories with help of EH's expertise. CL organized the dataset according to the defined subject categories. JL and EH did the descriptive statistics on the data and drafted the manuscript. JL wrote the final version. All authors carefully read the manuscript and approved of its publication.

## Funding

The authors were supported by an Open Research Area grant for the NESSHI Project, with funding from the Nederlandse Organisatie voor Wetenschappelijk Onderzoek (NWO) (CL) and the Deutsche Forschungsgemeinschaft (DFG) (JL and EH, project code: HI 1328/2-1).

### Conflict of interest statement

The authors declare that the research was conducted in the absence of any commercial or financial relationships that could be construed as a potential conflict of interest.

## Footnotes

^1^http://www.neurobioethics.org/ (accessed July 16, 2015).
