# Supplementary material for: Neuroethics 1995–2012. A Bibliometric Analysis of the Guiding Themes of an Emerging Research Field
Source: Front Hum Neurosci. 2016 Jul 1;10:336. doi: 10.3389/fnhum.2016.00336 (PMC4929847; doi:10.3389/fnhum.2016.00336)
Supplement: Supplementary file 1 [file Supplement1.DOCX]

# Supplement 1

**A: Procedure of subject-category formation:**

**B: Final Subject-categories by keywords**

|  | **Subject-category** | **Keyword** |
| --- | --- | --- |
|  | Addiction | abuse |
|  |  | addict* |
|  |  | alcohol |
|  |  | chronic |
|  |  | compulsion |
|  |  | craving |
|  |  | dependence |
|  |  | drug* |
|  |  | drug-seeking |
|  | Brain Death / Severe disorders of consciousness | brain dea* |
|  |  | brain injur* |
|  |  | coma* |
|  |  | death |
|  |  | end of life |
|  |  | head injur* |
|  |  | icu |
|  |  | intensive care |
|  |  | life sustaining |
|  |  | locked in |
|  |  | minimally conscious state |
|  |  | mcs |
|  |  | resuscitation |
|  |  | severe |
|  |  | suicide |
|  |  | terminal care |
|  |  | terminally |
|  |  | terminal disease |
|  |  | traumatic |
|  |  | vegetative state |
|  | Brain stimulation | dbs |
|  |  | deep brain stimulation |
|  |  | neuromodulation |
|  |  | stimulation |
|  |  | transcranial |
|  |  | tms |
|  | Enhancement | biomedical enhance* |
|  |  | cognitive enhance* |
|  |  | cognition enhancing |
|  |  | cosmetic neurology |
|  |  | human enhance* |
|  |  | moral enhance* |
|  |  | neuroenhance* |
|  |  | neurocognitive enhance* |
|  |  | pharmacological enhance* |
|  |  | psychopharmacological enhance* |
|  | Legal studies | court* |
|  |  | forensic |
|  |  | jail |
|  |  | judicial |
|  |  | jurisprudence |
|  |  | law |
|  |  | legal |
|  |  | legislature |
|  |  | responsibility |
|  | Medical research and medicine | ethics committee* |
|  |  | ethical behavior* |
|  |  | conflict* of interest |
|  |  | consent procedure* |
|  |  | formal consent |
|  |  | implied consent |
|  |  | incidental |
|  |  | informed consent |
|  |  | medical ethics |
|  |  | medical research |
|  |  | placebo |
|  | Moral Theory | autonomy |
|  |  | aggressi* |
|  |  | blame |
|  |  | conflict |
|  |  | consequentialis* |
|  |  | deontolog* |
|  |  | free will |
|  |  | moral agency |
|  |  | moral belief* |
|  |  | moral cognition* |
|  |  | moral decision |
|  |  | moral dilemma |
|  |  | moral emotion* |
|  |  | moral judgment* |
|  |  | moral mind |
|  |  | moral philosophy |
|  |  | moral psychology |
|  |  | moral value* |
|  |  | punishment |
|  |  | trolley problem |
|  |  | utilitarian |
|  |  | welfare |
|  | Molecular and genetic neuroscience | cell* |
|  |  | epigene* |
|  |  | epigenome |
|  |  | gene* |
|  |  | genome |
|  |  | molecular |
|  | Neuroimaging | Brain image* |
|  |  | CT |
|  |  | EEG* |
|  |  | diffusion tensor |
|  |  | fMRI |
|  |  | MRI |
|  |  | magnetic resonance imag* |
|  |  | neuroimaging |
|  |  | PET |
|  |  | scan |
|  | Neuroscience and society | communicating science |
|  |  | communicating scientific |
|  |  | coverage |
|  |  | cultur* |
|  |  | education* |
|  |  | policies |
|  |  | policy |
|  |  | policy recommendation |
|  |  | politic* |
|  |  | public |
|  |  | social |
|  |  | social implication* |
|  | Neurosurgery | BCI* |
|  |  | donat* |
|  |  | implant* |
|  |  | neurosurg* |
|  |  | psychosurg* |
|  |  | Surgery |
|  |  | surgic* |
|  |  | transplant* |
|  | Philosophy of mind and consciousness | brainhood |
|  |  | cartesian |
|  |  | conscious* |
|  |  | Descartes |
|  |  | embodied mind |
|  |  | epistemolog* |
|  |  | extended mind |
|  |  | instrumental |
|  |  | introspection |
|  |  | life world |
|  |  | mental state |
|  |  | mind |
|  |  | personhood |
|  |  | phenomen* |
|  |  | phenomenolog* |
|  |  | philosoph* |
|  |  | Qualia |
|  |  | rational |
|  |  | reduction |
|  |  | self-knowledge |
|  |  | subjective* |
|  |  | theory of mind |
|  | Psychiatric and neurodegenerative diseases and disorders | ADHD |
|  |  | Ageing |
|  |  | age related |
|  |  | Alzheimer* |
|  |  | Asperger* |
|  |  | attention deficit* |
|  |  | Autism |
|  |  | autist* |
|  |  | BIID |
|  |  | Bipolar |
|  |  | dement* |
|  |  | Electroconvulsive |
|  |  | epileps* |
|  |  | epilept* |
|  |  | mental disorder* |
|  |  | mental illness |
|  |  | nervous system disorder |
|  |  | Neurodegenerative |
|  |  | obsessive compulsive |
|  |  | Parkinson* |
|  |  | Psychiatry |
|  |  | psychopath* |
|  |  | Psychosis |
|  |  | schizo* |
|  | Psychopharmacology | antidepressant* |
|  |  | antiepileptic* |
|  |  | antipsychotic* |
|  |  | benzodiazepine |
|  |  | lorazepam |
|  |  | methylphenidate |
|  |  | modafinil |
|  |  | pharmaceut* |
|  |  | pharmaco* |
|  |  | propranolol |
|  |  | prozac |
|  |  | psychopharma* |
|  |  | psychotropic |
|  |  | SSRI* |
|  | Social and economic neuroscience | altruis* |
|  |  | antisocial |
|  |  | choice behavior |
|  |  | consumer* |
|  |  | cooperat* |
|  |  | decision-making |
|  |  | economics |
|  |  | emotion |
|  |  | empathy |
|  |  | financial |
|  |  | Game |
|  |  | happiness |
|  |  | incentive* |
|  |  | love |
|  |  | microeconomic* |
|  |  | nash |
|  |  | neuroeco* |
|  |  | pleasure |
|  |  | prosocial |
|  |  | reinforcement |
|  |  | reward* |
|  |  | social brain |
|  |  | social cognit* |
|  |  | social judgment* |
|  |  | social ties |
|  |  | trust |

**C: Content-based description of subject-categories**

|  | **Subject-category** | **Description** | **Example publication** |
| --- | --- | --- | --- |
|  | Addiction | - covers articles related to agency, drug abuse and the challenges addiction poses on concepts of free will and autonomy - includes publications addressing addiction from a social and psychological point of view - comprises work on the assumed potential of cognitive enhancing drugs to cause or at least to contribute to addiction | *Bell, Stephanie K., Lucke, J. C. and Hall, W. D. (2012) Lessons for enhancement from the history of cocaine and amphetamine use. AJOB Neuroscience, 3(2), p. 24-29*  *Goldstein, A. (2001) Addiction: from biology to drug policy, OUP, Oxford.* |
|  | Brain Death / Severe disorders of consciousness | - includes publications concerning the differentiation and diagnostics of brain death and minimally conscious states and the clinical disorders resulting in these states. - addresses ethical questions of intensive care and end-of-life decision making | *Freeman, E. A. (1997) Protocols for the vegetative state. Brain Inj, 11(11), p.837-49.*  *Varelas, P.N., Abdelhak, T., Hacein-Bey, L, (2008) Withdrawal of life-sustaining therapies and brain death in the intensive care unit. Semin Neurol, 28(5), p. 726-735.* |
|  | Brain stimulation | - addresses issues of technologies for brain stimulation in a general manner. - discusses clinical, technical and ethical issues of deep brain stimulation (dbs) and transcranial brain stimulation | *Kringelbach, M. L., Jenkinson, N., Green, A. L., et al. (2007) Deep brain stimulation for chronic pain investigated with magnetoencephalography, NeuroReport, 18(3), p.223-228.*  *Müller, S., Christen, M. (2011) Deep Brain Stimulation in Parkinsonian Patients? Ethical Evaluation of Cognitive, Affective, and Behavioral Sequelae, AJOB Neuroscience, 2(1), 3-13.* |
|  | Enhancement | - contains articles related to the effects of brain-technologies for cognitive or mood enhancement but not for treatment use - centers around the ethical and social discussions on the improvement of cognitive performance and on the biotechnical enhancement if humans in general | *Bublitz, J.C., Merkel, R., (2009) Autonomy and authenticity of enhanced personality traits. Bioethics, 23(6), p.360-374.*  *Chatterjee, A. (2004) Cosmetic neurology: The controversy over enhancing movement, mentation, and mood. Neurology, 63(6), p. 968-74.* |
|  | Legal studies | - includes studies on the implications of neurobiological and psychological investigations into the nature of free will for the legal system - comprises studies on the legal aspects of clinical research ethics and end-of-life situations | *Knabb, J.J., Welsh, R.K., Ziebell, J.G., Reimer, K.S. (2009) Neuroscience, moral reasoning, and the law. Behav Sci Law, 27(2), p. 219-236.*  *Kulynych, J.J. (2002) Legal and ethical issues in neuroimaging research: human subjects protection, medical privacy, and the public communication of research results. Brain and Cognition, 50(3), p.345-357.* |
| 1. - | Medical research and medicine | - comprises publications dealing with ethical questions relating to the practice of clinical research and patient treatment in the neurosciences such as incidental findings or informed consent | *Eijkholt, M., Anderson, J. A., Illes, J., (2012) Picturing neuroscience research through a human rights lens: imaging first-episode schizophrenic treatment-naive individuals. Int J Law Psychiatry, 35(2), p. 146-152.*  *Illes, J.,, Reimer, J.C., Kwon, B.K., (2011) Stem Cell Clinical Trials for Spinal Cord Injury: Readiness, Reluctance, Redefinition. Stem Cell Re, 7(4), p. 997-1005* |
|  | Moral Theory | - addresses the psychology and neurobiology of moral-decision making - focusses on questions of determinism and free-will - questions the function of moral theory in neurosciences. - discusses challenges to established interpretations of morally significant concepts such as autonomy, responsibility and human nature. | *Bruni, T. (2012) Ventromedial Prefrontal Cortex Lesions and Motivational Internalism. AJOB Neuroscience, 3(3), p. 19-23.*  *Nichols, S. (2004) After objectivity: an empirical study of moral judgment. Philosophical Psychology, 17 (1), p. 3-26.* |
|  | Molecular and genetic neuroscience | - focusses on the epidemiology and molecular biology of psychiatric and neurodegenerative diseases or addiction - questions ethical decision-making in molecular and genetic neuroscience | *Kocerha, J., Kauppinen, S., Wahlestedt, C. (2009) microRNAs in CNS Disorders. NeuroMolecular Medicine, 11(3), p. 162-172.*  *Schneider, S.A., Schneider, U.H., Klein, C.A. (2011) Genetic testing for neurologic disorders. Semin Neurol, 31(5), p. 542-552.* |
|  | Neuroimaging | - includes all publications referring to the methodology of neuroimaging regardless of the overall topic addressed. | *Kozel, F.A., Johnson, K.A., Mu, Q., Grenesko, E.L., et al. (2005) Detecting deception using functional magnetic resonance imaging. Biological Psychiatry, 58(8), p. 605-613.*  *Gregory, S., Ffytche, D., Simmons, A., Kumari, V., et al. (2012) The Antisocial Brain: Psychopathy Matters: A Structural MRI Investigation of Antisocial Male Violent Offenders. Arch Gen Psychiatr, 69(9), p. 962-972.* |
|  | Neuroscience and society | - includes articles on the interfacing between neurosciences and the public - comprises publications on the awareness of the neurosciences and neuroethics in the public and in the media - contains work on ethical issues of policy making with regard to neuroscience research and its applications in society | *Illes, Judy, Moser, M. A., McCormick, Jennifer B,, Racine, Eric, et al. (2010) Neurotalk: improving the communication of neuroscience research. Nat Rev Neurosc, 11, p.61-69*  *Coveney, C. M., Nerlich, B., Martin, P., (2009) Modafinil in the media: metaphors, medicalisation and the body. Soc Sci Me, 68(3), 487-495* |
|  | Neurosurgery | - comprises work on neurosurgical innovation and on neural grafting - does not include related philosophical issues such as questions of personal identity with regard to brain surgery | *Galpern, W.R., Corrigan-Curay, J., Lang, A.E., et al. (2012) Sham neurosurgical procedures in clinical trials for neurodegenerative diseases: scientific and ethical considerations. Lancet Neurol, 11(7), p. 643-650.*  *Gillett, G.R., Honeybul, S., Ho, K.M., Lind, C.R., (2010) Neurotrauma and the RUB: where tragedy meets ethics and science. J Med Ethics, 36(12), p. 727-730.* |
|  | Philosophy of mind and consciousness | - includes articles discussing theories of consciousness, the problems of other minds and in general the possibility of mind in a material world with relation to research from cognitive science | *Levy, D.A. (2003) Neural holism and free will. Philosophical Psychology, 16(2), p. 205-226.*  *Northoff, G. (2012) From emotions to consciousness - a neuro-phenomenal and neuro-relational approach. Front Psychol, 3:303.* |
|  | Psychiatric and neurodegenerative diseases and disorders | - focus on neuroethics as the ethics relating to care for pathological conditions. - special attention given to the questions of free will, responsibility and informed consent in mental illness as well as the underlying neurobiological mechanisms of psychiatric disorders. | *Riepe, M.W., Mittendorf, T., Förstl, H., et al. (2009) Quality of Life as an outcome in Alzheimer's disease and other dementias - obstacles and goals. BMC Neurol, 9(47).*  *Keefe, R.S., Goldberg, T.E., Harvey, P.D., Gold, J.M. et al. (2004) The Brief Assessment of Cognition in Schizophrenia: reliability, sensitivity, and comparison with a standard neurocognitive battery. Schizophr Re, 68(2-3), p. 283-297.* |
|  | Psychopharmacology | - covers articles on the effect of pharmacological substances for treatment and on the effects of drug (ab-)use in the brain - addresses the role of the pharmaceutical industry in the (neuro-)ethics discourse. - comprises work on the pharmacological treatment of neurological and mental illnesses and the ethical issues of clinical research in psychopharmacology | *Andrews, W., Parker, G., Barrett, E. (1998) The SSRI antidepressants: exploring their "other" possible properties. J Affect Disor, 49(2), p.141-144.*  *Wasserman, D.T., Liao, S.M., (2008) Issues in the Pharmacological Induction of Emotions. Journal of Applied Philosophy 25(3), 178-192.* |
|  | Social and economic neuroscience | - includes publications analyzing discourses on the handling of neurological and psychiatric diseases from a sociological perspective - comprises analysis of research policy changes with relation to neuroscience. - discusses decision-making processes in economic contexts with a focus on current research from a neuroscience and cognitive science perspective. | *Kable, J.W., Glimcher, P.W. (2007) The neural correlates of subjective value during intertemporal choice. Nat Neurosc, 10(12), p.1625-1630.*  *Adolphs, R . (2003) Cognitive Neuroscience of Human Social Behaviour. Nature Reviews Neuroscience, 4(3), p.165-178.* |

**D: Terms with the highest tf-idf value per category**

|  | **Category** | **Term (tf-idf)** |
| --- | --- | --- |
|  | Addiction | alcohol (29.4)  addiction (29.149351)  alcohol dependent group (9.0)  gambler (9.0)  drug (8.139535)  addictive (6.095238)  heroin (6.0)  mmt (6.0)  substance abuse (5.0625)  drug neuroethics (4.5)  willpower (4.5)  addiction aim (4.5)  craving (4.1666665)  slowed (4.0)  methadone (4.0)  drug alcohol (4.0)  21 male (4.0)  illicit (4.0)  pomc (4.0)  leptin (4.0)  addicted to food (4.0)  treating drug (4.0)  reevaluation (4.0)  obesity have addiction (4.0)  vaccine (3.7692308) |
|  | Brain Death / Severe disorders of consciousness | death (97.31579)  brain death (72.25)  resuscitation (64.0)  hasting (36.0)  brain injury (27.008928)  anesth (25.0)  organ donation (23.676056)  vegetative (22.74436)  life sustaining (19.862068)  donor (17.066668)  tomography (16.333334)  severely brain (16.0)  brain death in adult (16.0)  traumatic brain injury (15.754098)  withdrawal (14.58)  coma (13.884615)  palliative (12.461538)  prognosis (12.15)  death dcd (12.0)  life care (11.56)  minimally conscious (11.445544)  physician assisted (10.666667)  brain dead (10.666667)  tbi (10.125)  euthanasia (10.027778) |
|  | Brain Stimulation | deep brain stimulation (106.5)  dbs (105.020836)  tdc (56.25)  deep brain stimulation dbs (36.75168)  direct current stimulation tdc (24.5)  stimulation tdc (24.5)  transcranial magnetic stimulation (20.828571)  tms (16.487804)  stn (13.795918)  refractory (13.432836)  psychiatric (11.244147)  electrode (9.68)  parkinson disease (9.6)  subthalamic nucleus (9.256411)  obsessive compulsive disorder (9.152381)  transcranial direct (9.142858)  transcranial direct current stimulation (9.142858)  erregbarkeit (9.0)  lateral sclerosis (9.0)  zusammenfassung (9.0)  methoden (9.0)  par (9.0)  phantom limb pain (9.0)  paradoxe (9.0)  excitability (9.0) |
|  | Enhancement | enhancement (83.56626)  cognitive enhancement (50.46)  neuroenhancement (33.517242)  enhancer (29.641026)  cognitive (25.940752)  neurocognitive enhancement (21.043478)  moral enhancement (16.40909)  ethics of cognitive (16.0)  enhancing (14.797203)  ne (12.8)  neuroenhancement ne (12.5)  cognitive enhancer (11.523809)  enhancing drug (11.25)  drug (10.810811)  neurocognitive (10.204724)  net wt (10.0)  pharmacological (9.82313)  cosmetic neurology (9.8)  aspekte (9.0)  work therapy (9.0)  neurocognitive enhancement therapy (9.0)  unbidden (9.0)  atypical antipsychotic (9.0)  mastery (9.0)  unnaturalness (9.0) |
|  | Legal Studies | law (80.19802)  desire for limb amputation (36.0)  bodied (36.0)  forensic (22.773195)  ethical (19.747288)  neuroscience (19.46768)  left sidedness (16.0)  supreme court (16.0)  somatosensory disturbance (16.0)  criminal responsibility (15.428572)  criminal law (15.0)  limb amputation (12.0)  death (9.285024)  subclinical (9.0)  mermer (9.0)  efm (9.0)  ngland academy of clinical (9.0)  nglandn (9.0)  ngland (9.0)  pardo (9.0)  carer (9.0)  academy of clinical neuropsychology (9.0)  event in question (9.0)  diagnosed with disorder (9.0)  religiosity (9.0) |
|  | Medical research and medicine | informed consent (74.141365)  incidental finding (49.877777)  placebo (45.066666)  ethical (40.04885)  ethics (36.661274)  medical ethics (23.076923)  clinical (22.150843)  neuroscience nursing (16.0)  ethics committee (15.157895)  medical school (12.5)  disclosure (12.448276)  moral duty (12.0)  neuroradiologist (11.645162)  mri (9.958621)  depressionen (9.0)  working with older (9.0)  implied consent (9.0)  incidentalome (9.0)  cs (9.0)  postgraduate neurology (9.0)  city (9.0)  hyperthermia (9.0)  neuroscience nurse (9.0)  neuroclinical (9.0)  older person (9.0) |
|  | Molecular Neurobiology and Genetics | embryonic (17.285715)  molecular (14.880953)  neural crest (12.0)  stem cell (11.593103)  cellular (10.451612)  primate stroke (9.0)  gene regulatory (9.0)  mol (9.0)  nc (9.0)  mirnas (8.0)  genome (7.6923075)  progenitor (5.3333335)  rna (5.3333335)  embryonic stem cell (4.6538463)  rat brain (4.5)  molecule (4.5)  micrornas mirnas (4.5)  transient (4.5)  neural crest stem cell (4.5)  embryonic tissue (4.0)  human nc cell (4.0)  cellular and molecular (4.0)  micrornas (4.0)  rna molecule (4.0)  human medial temporal lobe (4.0) |
|  | Moral Theory | moral judgment (97.98387)  compatibilist (64.0)  lsquo (49.0)  rsquo (49.0)  crescioni (36.0)  incompatibilist (36.0)  alquist (36.0)  impersonal moral (36.0)  haidt (36.0)  moral decision (35.161617)  behavior (29.886793)  utilitarian (29.76087)  neural (29.288918)  baumeister (27.0)  emotional (23.52)  neuroscience (22.986942)  moral cognition (22.272728)  dilemmas (20.346153)  animal welfare (20.25)  cortex (19.88499)  human moral (19.692308)  moral dilemmas (19.6)  aggression (18.775862)  personal moral (18.75)  psychology (18.127167) |
|  | Neuroimaging | imaging (227.35068)  neuroimaging (191.16347)  fmri (101.929245)  cortex (101.50244)  activation (87.38298)  magnetic resonance imaging (84.8984)  stimulation (83.565216)  neural (75.001465)  thalamic (64.0)  mri (60.009617)  brain imaging (57.188118)  prefrontal cortex (45.733334)  scan (45.63158)  medial (44.32787)  incidental finding (44.04301)  parietal (40.015385)  neuroscience (37.431637)  gyrus (35.851852)  cingulate (31.25)  insula (30.153847)  brain image (28.8)  orbitofrontal (27.675676)  emotional (26.162163)  neural activation (25.0)  synchronization (25.0) |
|  | Neuroscience and society | public (59.05899)  cheapening (36.0)  psychoactive (18.0)  ethical (17.635628)  neuroscience (17.46334)  ethics (16.429422)  simplistic (16.0)  steven spielberg (16.0)  cultural (14.159292)  educator (13.5)  coverage (12.970589)  interactive (12.5)  worldview (9.090909)  neurobiology of intelligence (9.0)  embryonic stem (9.0)  neuroscience society (9.0)  classroom (9.0)  brain and culture (9.0)  ethics service (9.0)  director (9.0)  ethical discourse (9.0)  cardiac (9.0)  memetic (9.0)  humanist (9.0)  dependency (9.0) |
|  | Neurosurgery | transplantation (49.20714)  stem cell (37.72269)  refractory psychiatric (36.0)  surgery (32.839622)  deep brain stimulation (28.789167)  dbs (28.45889)  surgical (28.0)  donor (26.74074)  embryonic (25.0)  ganglia (25.0)  mesenchymal stem cell (25.0)  death (24.252632)  organ donation (23.676056)  neurosurgical (20.378948)  ethical (18.579351)  neurosurgery (16.118422)  autologous adult (16.0)  transplant (15.612904)  therapy (15.0865135)  tissue (14.083333)  magnetic (12.5)  cardiac death (11.645162)  implantation (11.636364)  neurosurg (11.255814)  dcd (11.172414) |
|  | Philosophy of Mind and Consciousness | severe brain (75.0)  awareness (61.12371)  phenomenology (50.0)  memoir (36.0)  phenomenological (28.9)  disorder of consciousness (28.40909)  dialogical (25.0)  philosophical (24.832512)  vegetative (20.807407)  conscious cogn (20.166666)  reductionism (18.0)  neuroscience (17.46334)  minimally conscious (16.842106)  vm (16.0)  automated (16.0)  memoir writing (16.0)  cartesian (16.0)  synthese (16.0)  descarte (16.0)  enlightenment (16.0)  spiritual experience (16.0)  sociality (16.0)  cognitive (12.800769)  naturalism (12.5)  mind and machine (12.5) |
|  | Psychiatric and neurodegenerative diseases and disorders | alzheimer (832.0)  disease pd (324.0)  parkinson (284.4878)  advocacy (169.0)  psychiatric (142.37038)  dementia (141.512)  disease ad (130.66667)  psychiatry (109.70149)  schizophrenia (97.31645)  clinical (83.19358)  impairment (79.08497)  deficit (73.78723)  cognitive (65.53293)  patienten mit (64.0)  strungen (64.0)  replacement therapy (64.0)  advocacy website (64.0)  ethical (63.64449)  adhd (60.166668)  symptom (57.18919)  dbs (54.335403)  alzheimer disease (54.258064)  therapy (53.440475)  prefrontal (52.403225)  nucleus (52.07143) |
|  | Pychopharmacology | drug (47.162792)  aed (40.5)  antidepressant (31.114286)  pharmacological (30.008064)  enhancement (28.050434)  pharmaceutical (27.472527)  enhancer (27.225)  psychotropic (16.666666)  pregnant women (16.0)  antiepileptic drug aed (16.0)  maternal separation (16.0)  maternally separated (16.0)  running wheel (16.0)  cognitive (15.667443)  antipsychotic (13.793103)  psychopharmacology (13.787234)  psychopharmaka (13.5)  medication (12.030075)  antiepileptic drug (12.0)  psychopharmaceutical (12.0)  neurocognitive (11.552)  psychopharmacological (11.307693)  cognitive enhancement (10.546875)  ethical (9.90753)  ssris (9.8) |
|  | Social and Economic neuroscience | empathy (21.460318)  mirror neuron (21.125)  monkey (20.25)  neuroeconomic (20.055555)  behavior (18.449919)  mind society (16.0)  social behaviour (16.0)  society abstract nbsp nbsp (16.0)  organisation (16.0)  mind society abstract nbsp (16.0)  neural (15.478643)  cortex (15.087619)  altruistic (15.0)  prefrontal cortex (12.444445)  emotional (11.111111)  altruism (11.076923)  empathic (10.125)  love (10.0)  social cognition (9.346154)  substrate (9.03125)  social behavior (9.025)  imitator (9.0)  feeling of engagement (9.0)  equilibrium (9.0)  risky decision (9.0) |

**E: Calculation of the connectivity of subject categories**

$i_{a,b}$ is a measure for the intensity of the connection between two topical subject-categories $a$ and $b$:

$n_{a,b}$ = number of bibliographical references which have been categorized as belonging both to subject-categories $a$ and $b$

$n_{a}$ = total number of bibliographical references categorized in $a$

$n_{b}$= total number of bibliographical references categorized in $b$

$$i_{a,b}=\frac{n_{a,b}}{n_{a}\times n_{b}}$$

In practice, the values $i_{a,b}$ are too small to be visualized in Gephi, the network visualization software used to produce figure 4. Therefore, we multiplied the intensities of all connections by a constant c, for the purpose of scaling them to a range of values $w$ (for weight, the term used in network analysis for the strength of a connection).

$m$ = number of bibliographical references in the topic which has the maximum number of references (in practice: $m = 632$).

$$c = m \times1000000$$

$${w=i}_{a,b}\times c$$
