# Supplementary material for: Neuroethics 1995–2012. A Bibliometric Analysis of the Guiding Themes of an Emerging Research Field
Source: Front Hum Neurosci. 2016 Jul 1;10:336. doi: 10.3389/fnhum.2016.00336 (PMC4929847; doi:10.3389/fnhum.2016.00336)
Supplement: Supplementary file 2 [file Supplement2.DOCX]

**Supplement 2**

**Relevant Journals in Neuroethics and their category**

|  | **Journal** | **Abbr.** | **Journal Category** | **ISI-Classification** |
| --- | --- | --- | --- | --- |
|  | Academic Psychiatry | acad psychiatry | Biomed | Medicine / Social Science |
|  | Accountability in Research | account res | SSH | Social Science |
|  | Acta Psychiatrica Scandinavica | acta psychiatr scand | biomed | Medicine |
|  | Addictive Behaviours | addict behav | biomed | Medicine |
|  | Addiction | addiction | biomed | Medicine |
|  | American Journal of Neuroradiology | am j neuroradiol | biomed | Medicine |
|  | American Journal of Bioethics | am j bioethics | SSH | Medicine |
|  | American Journal of Bioethics - Neuroscience | ajob neuroscience | SSH | Neuroscience |
|  | Anesthesia & Analgesia | anesth analg | biomed | Medicine |
|  | Annals of the New York Academy of the Sciences | ann n y acad sci | biomed | Biochemistry |
|  | Archives of General Psychiatry | arch gen psychiatry | biomed | Medicine |
|  | Aviation, Space and Environmental Medicine (i.e. Aviation, Space and Human Performance) | ashp | biomed | Medicine |
|  | Behavioural Science & the Law | behav sci law | SSH | Medicine |
|  | Bioethics | bioethics | SSH | Arts and Humanities, Medicine, Social Science |
|  | British Journal of Anaestesia | br j anaesth | biomed | Medicine |
|  | Brain & Cognition | brain cogn | biomed | Psychology |
|  | Brain Stimulation | brain stimul | biomed | Biochemistry |
|  | Cambridge Quarterly of Healthcare Ethics | camb q healthc ethic | SSH | Medicine, Social Science |
|  | Cerebral Cortex | cereb cortex | biomed | Neuroscience |
|  | Cerebrum | cerebrum | biomed | Medicine, Neuroscience |
|  | Clinical Neurophysiology | clin neurophysiol | biomed | Medicine, Neuroscience |
|  | Clinical Pharmacology & Therpeutics | clin pharmacol ther | biomed | Medicine |
|  | Consciousness and Cognition | conscious cogn | biomed | Arts and Humanities, Psychology |
|  | Cortex | cortex | biomed | Arts and Humanities, Medicine, Neuroscience |
|  | Critical Care Medicine | crit care med | biomed | Medicine |
|  | Current Opinion in Neurology | curr opin neurol | biomed | Medicine, Neuroscience |
|  | Current Opinion in Psychiatry | curr opin psychiatr | biomed | Medicine |
|  | Der Nervenarzt | der nervenarzt | biomed | Medicine, Neuroscience |
|  | Ethik in der Medizin | ethik med | SSH | Arts and Humanities, Medicine, Social Science |
|  | European Archives of Psychiatry and Clinical Neuroscience | eur arch psychiatry clin neurosci | biomed | Medicine, Neuroscience |
|  | Fortschritte in Neurologie und Psychiatrie | fortschr neurol psyc | biomed | Medicine, Neuroscience |
|  | Frontiers in integrative neuroscience | front integr neurosci | biomed | Neuroscience |
|  | Frontiers in human neuroscience | front hum neurosci | biomed | Medicine, Neuroscience, Psychology |
|  | Hastings Center Report | hastings cent rep | SSH | Arts and Humanities, Medicine, Social Science |
|  | Human brain mapping | hum brain mapp | biomed | Medicine, Neuroscience |
|  | International Congress Series | int congr ser | biomed | Medicine, Immunology |
|  | International Journal of Law and Psychiatry | int j law psychiatry | SSH | Medicine, Social Science |
|  | International Journal of Neuropsychopharmacology | int j neuropsychop | biomed | Medicine, Pharmacology |
|  | International Series on Technology, Policy and Innovation | int s techn pol inn | SSH | -- |
|  | Journal of the American Academy of Psychiatry and the Law | j am acad psychiatry law | SSH | Medicine |
|  | Journal of Bioethical Inquiry | j bioethic inq | SSH | Medicine, Social Science |
|  | Journal of Clinical Ethics | j clin ethics | SSH | Medicine, Social Science |
|  | Journal of Clinical Neuroscience | j clin neurosci | biomed | Medicine, Neuroscience |
|  | Journal of Cognitive Neuroscience | j cogn neurosci | biomed | Arts and Humanities, Neuroscience, Social Science |
|  | Journal of Law, Medicine & Ethics | j law med ethics | SSH | Medicine |
|  | Journal of Medical Ethics | j med ethics | SSH | Arts and Humanities, Medicine, Social Science |
|  | Journal of Neurology | j neurol | biomed | Medicine, Neuroscience |
|  | Journal of Neurosurgery | j neurosurg | biomed | Medicine |
|  | Journal of Trauma and Acute Care Surgery | j trauma | biomed | Medicine |
|  | Journal of the American Medical Association | JAMA | biomed | Medicine |
|  | Journal of Applied Philosophy | journal of applied philosophy | SSH | Arts and Humanities |
|  | Medical Hypotheses | med hypotheses | biomed | Medicine |
|  | Medicine, Health Care and Philosophy | med health care philos | SSH | Medicine, Social Science |
|  | Mind & Society | mind & society | SSH | Arts and Humanities, Social Science |
|  | Minds and Machines | mind mach | biomed | Arts and Humanities, Computer Science |
|  | Molecular Psychiatry | mol psychiatry | biomed | Biochemistry, Medicine, Neuroscience |
|  | Nature | nature | biomed | Multidisciplinary |
|  | Nature Neuroscience | nat neurosci | biomed | Neuroscience |
|  | Nature Reviews Neuroscience | nat rev neurosci | biomed | Neuroscience |
|  | Neurobiology of Aging | neurobiol aging | biomed | Medicine, Neuroscience, Biochemistry |
|  | Neurocritical Care | neurocrit care | biomed | Medicine |
|  | Neuroethics: defining the issues in theory, practice and policy |  | SSH | -- |
|  | Neuroethics: mapping the field; conference proceedings |  | SSH | -- |
|  | Neuroethics | neuroethics | SSH | Medicine, Neuroscience |
|  | Neuroimage | neuroimage | biomed | Neuroscience |
|  | Neurological Sciences | neurol sci | biomed | Medicine |
|  | Neurology | neurology | biomed | Arts and Humanities, Medicine |
|  | Neuromodulation | neuromodulation | biomed | Medicine, Neuroscience |
|  | NeuroMolecular Medicine | neuromol med | biomed | Neuroscience, Biochemistry |
|  | Neuron | neuron | biomed | Neuroscience |
|  | Neuropsychology Review | neuropsychol rev | biomed | Psychology |
|  | Neuropsychologia | neuropsychologia | biomed | Arts and Humanities, Neuroscience, Psychology |
|  | Neuroscience Research | neurosci res | biomed | Neuroscience |
|  | Neurosurgical Focus | neurosurg focus | biomed | Medicine |
|  | Neurotherapeutics | Neurotherapeutics | biomed | Medicine, Pharmacology |
|  | Proceedings of the National Academy of the Sciences (PNAS) | p natl acad sci usa | biomed | Multidisciplinary |
|  | Perspectives in Biology and Medicine | perspect biol med | biomed | Arts and Humanities, Medicine |
|  | Phenomenology and the Cognitive Sciences | phenomenol cogn sci | SSH | Arts and Humanities, Neuroscience |
|  | Philosophical Psychology | philos psychol | SSH | Arts and Humanities, Psychology |
|  | Philosophical Transactions of the Royal Society B: Biological Sciences | phil trans r soc b | biomed | Agricultural and Biological Sciences |
|  | PLoS Biology | plos biol | biomed | Agricultural and Biological Sciences |
|  | PLoS One | plos one | biomed | Agricultural and Biological Sciences, Medicine |
|  | Poiesis & Praxis | poiesis & praxis | SSH | Engineering, Social Sciences |
|  | Progress in Brain Research | prog brain res | biomed | Neuroscience |
|  | Psychiatric Clinics of North America | psychiatr clin north am | biomed | Medicine |
|  | Psychiatric Services | psychiatr serv | biomed | Medicine |
|  | Psychiatrie und Psychotherapie | psychiatrie und psychotherapie | biomed | Medicine |
|  | Radiology | radiology | biomed | Medicine |
|  | Review of Philosophy and Psychology | rev phil psych | SSH | Arts and Humanities, Psychology |
|  | Schizophrenia Bulletin | schizophr bull | biomed | Medicine |
|  | Schizophrenia Research | schizophr res | biomed | Medicine |
|  | Scientific American | sci am | biomed | Multidisciplinary |
|  | Science and Education | sci educ | SSH | Social Sciences |
|  | Science and Engineering Ethics | sci eng ethics | SSH | Business and Management, Social Sciences, Medicine |
|  | Social Cognitive and Affective Neuroscience | soc cogn affect neurosci | biomed | Neuroscience, Psychology |
|  | Social Neuroscience | soc neurosci | biomed | Neuroscience, Psychology, Social Sciences |
|  | Social Science & Medicine | soc sci med | SSH | Arts and Humanities, Social Sciences, Medicine |
|  | Sociology of Health and Illness | sociol health illn | SSH | Social Sciences, Medicine |
|  | Synthese | Synthese | SSH | Arts and Humanities, Social Sciences |
|  | Theoretical Medicine and Bioethics | theor med bioeth | SSH | Medicine |
|  | Trends in Cognitive Science | trends cogn sci | biomed | Neuroscience, Psychology |
|  | Update in Intensive Care and Emergency Medicine | upd int car | biomed | Medicine |
|  | World Neurosurgery | world neurosurg | biomed | Medicine |
