# Supplementary material for: Neuroethics 1995–2012. A Bibliometric Analysis of the Guiding Themes of an Emerging Research Field
Source: Front Hum Neurosci. 2016 Jul 1;10:336. doi: 10.3389/fnhum.2016.00336 (PMC4929847; doi:10.3389/fnhum.2016.00336)
Supplement: Supplementary file 3 [file Supplement3+4.DOCX]

**Supplement 3**

**Training and affiliations of MND top neuroethics researchers**

| **Name** | **Initial training** | **Highest Academic Degree** | **Current affiliation** | **Professor/ Researcher of…** | **Country of host institution** | **Top Author in the categories of…** |
| --- | --- | --- | --- | --- | --- | --- |
| Adrian Carter | ? | PhD | UQ Centre for Clinical Research, The University of Queensland | Addiction neuroethics | Australia | Addiction |
| Adrian Raine | Psychology | PhD | University of Pennsylvania, Department of Criminology | Neurobiology of antisocial behavior | US | Legal Studies, Neuroimaging, Social Neuroscience |
| Ahmed Dahir Mohamed | Psychology | PhD | Department of Psychology, University of Nottingham | Post-Doctoral Fellow in Psychology (Cognitive Neuroscience) | UK | Neuroscience and Society |
| Alan G. Sanfey | Cognitive Psychology | PhD | Department of Psychology, University of Arizona | Associate Professor | US | Social Neuroscience |
| Alexander Kurz | Medical Doctor | MD + PhD | Neurochemistry and Neurogenetics Laboratory, Department of Psychiatry and Psychotherapy, Technische Universität München | Oberarzt | Germany | Molecular Neurobiology and Genetics, Psychiartic and neurodegenerative diseases |
| Allyson C. Rosen | Psychology | PhD | Veteran's Administration , Palo Alto Health Care System and Stanford University School of Medicine | ? | US | Neuroscience and Society |
| Alvaro Pascual-Leone | Medical Doctor | MD + PhD | Berenson-Allen Centre for Non-Invasive Brain Stimulation, Harvard University | Professor of Neurology | US | Brain Stimulation |
| Anders Sandberg | Computational Neuroscience | PhD | James Martin Research Fellow at the Future of Humanity Institute, Faculty of Philosophy, University of Oxford | Research Fellow at the Future of Humanity Institute | UK | Enhancement |
| Andreas G Franke | Medical Doctor, Social Science | MD + PhD | University Medical Centre, Mainz | Psychiatry | Germany | Addiction, Enhancement |
| Andres M Lozano | Medical Doctor | MD + PhD | Division of Brain Imaging & Behaviour Systems - Neuroscience, Toronto Western Research Institute (TWRI | Dan Family Chair in Neurosurgery, University of Toronto | Canada | Brain Stimulation, Neurosurgery |
| Andrew Fenton | Philosophy | PhD | Department of Philosophy, Fresno State University | Lecturer | US | Psychopharmacology |
| Anjan Chatterjee | Medical Doctor, Philosophy | MD + BA | Center for Neuroscience and Society at the University of Pennsylvania | Professor of Neurology | US | Enhancement |
| Anthony Phillips | Psychology | PhD | Canadian Institute of Health Research, Institute of Neuroscience Mental Health and Addiction | Professor of Psychiatry, University of British Columbia, INMHA Scientific Director | Canada | Neuroscience and Society |
| Antoine Bechara | Neuroscience, Psychology | PhD | Department of Psychology, University of Southern California | Addiction Nueroscience | US | Addiction |
| Archie Alexander | Medical Doctor + Law | MD + JD + LLM | Faculty of Law, Thammasat University, Bangkok | Independent Consultant for Health Law, Policy and Bioethics | Thailand | Psychopharmacology |
| Arthur L Caplan | Philosophy | PhD | Division of Bioethics at New York University Langone Medical Centre | Bioethics | US | Addiction, Neuroscience and Society |
| Barbara J Sahakian | Experimental Psychology | PhD | Cambridge University, Department of Psychiatry | Neuropsychiatry | UK | Medical Research, Addiction, Brain Death, Enhancement, Psychiatric and Neurodegenerative Diseases, Psychopharmacology, Neuroscienc and Socity |
| Bert De Smedt | Educational Science | PhD | Parenting and Special Education Research Group, KU Leuven | Assistant professor in Educational Neuroscience | Belgium | Neuroscience and Society |
| Betty J. Pfefferbaum | Medical Doctor | MD | Department of Psychiatry and Behavioral Sciences, College of Medicine, University of Oklahoma Health Sciences Center, Oklahoma City | Psychiatrist | US | Neuroscience and Society |
| Bruce E. Wexler | Medical Doctor | MD | Yale University School of Medicine | Professor Emeritus of and Senior Research Scientist in Psychiatry | US | Psychiatric and neurodegenerative diseases |
| C J Barrios | ? | ? | Department of Neurosurgery, Hospital of the University of Pennsylvania, Philadelphia | Neurosurgery | US | Addiction |
| Carla L. Harenski | Neuroscience | PhD | The Mind Research Network, Albuquerque | Assistant Professor of Translational Neuroscience | US | Neuroimaging |
| Caroline Bonertz | Medical Doctor | MD | Katholisches Klinikum Mainz | Medical Doctor | Germany | Addiction, Enhancement |
| Colin A Klein | Philosophy | PhD | Department of Philosophy, Macquarie University, Sydney | Associate Professor of Philosophy | Australia | Legal Studies |
| Corinna Porteri | Philosophy (Bioethics) | PhD | Bioethics Unit at the IRCCS Saint John of God Fatebenefratelli Brescia, Italy | Bioethics | Italy | Brain Death |
| CR Lind | ? | ? | Centre for Neuromuscular and Neurological Disorders, University of Western Australia | ? | Australia | Brain Death |
| Craig Nicholson | Biochemistry | BSc | MA Healthcare | Editor, International Journal of Palliative Nursing and British Journal of Neuroscience Nursing | UK | Neuroscience and Society |
| Cynthia Forlini | Neurological Sciences | PhD | University of Queensland, UQ center for clinical research | Neuroethics | Australia | Enhancement, Neuroscience and Society |
| D Manthey | ? | ? | ? | ? | ? | Molecular Neurobiology and Genetics |
| Dan J. Stein | Medical Doctor + Philosophy | MD + 2 PhD | Department of Psychiatry and Mental Health, University of Cape Town | Professor and Chair of Department | South Africa | Psychopharmacology |
| Dan Larriviere | Medical Doctor + Attorney | MD + JD | Acting Chair, Department of Neurology, Ochsner Neuroscience Institute, New Orleans | Neurologist | US | Enhancement, Legal Studies |
| Daniel Ansari | Psychology, Neuroscience | PhD | Numerical Cognition Laboratory, Department of Psychology, University of Western Ontario | Professor of Psychology | Canada | Philosophy of Mind and Consciousness, Neuroscience and Society |
| Daniel D Langleben | Medical Doctor | MD | Department of Psychiatry, Perlman Medical School, University of Pensylvania | Psychiatry | US | Medical Research, Legal Studies |
| Daniel R Weinberger | Medical Doctor, Neuroscientist | MD + PhD | Clinical Studies Section, NIH, Bethesda | Chief of the Clinical Brain Disorders Branch | US | Molecular Neurobiology and Genetics |
| Daniel Z Buchman | Medical Ethics, Social Medicine | PhD | Joint Centre for Bioethics, University of Toronto | Medical Ethics | Canada | Addiction, Neuroscience and Society |
| Danielle C Turner | Neuroscience | PhD | Post-Doc at University of Cambridge Department of Psychiatry | ? | UK | Enhancement |
| David M Blass | Medical Doctor + Jewish Theology | MD + Rabbi | Department of Psychiatry and Behavioral Sciences, Johns Hopkins University, Baltimore | Associate Professor | US | Molecular Neurobiology and Genetics |
| David R. Thorne | ? | ? | Department of Neurobiology and Behavior, Division of Neuropsychiatry, Walter Reed Army Institute of Research | ? | US | Psychopharmacology |
| Davor Solter | Medical Doctor, Developmental Biologist | MD + PhD | Institute of Medical Biology, A*STAR, National University of Singapore, Singapore | ? | Singapore | Molecular Neurobiology and Genetics |
| Dawn Bowers | Clinical Psychology | PhD | Centre for Movement Disorders and Neurorestauration, University of Florida | Professor in Clinical and Health Psychology, Neurology | US | Brain Stimulation, Neurosurgery, Psychiatric and neurodgenerative diseases |
| Debra J H Mathews | Molecular Biology + Bioethics | PhD + MA | Barman Institute of Bioethics, Johns Hopkins University Baltimore | Assistant Director for Science Programs | US | Molecular Neurobiology and Genetics, Neurosurgery |
| Dominic Wilkinson | Medical Doctor | MD + PhD | Oxford Uehiro Centre for Practical Ethics | Medical Ethics | UK | Brain Death |
| Douglas Kerr | Medical Doctor, Molecular Biology | MD, PhD | Johns Hopkins Transverse Myelopathy Center, Johns Hopkins University, Baltimore | Associate Professor | US | Molecular Neurobiology and Genetics |
| Elaine Snell | ? | ? | European Dana Alliance for the Brain, Snell Communications Ltd, Science, Medicine and Health / British Neuroscience Association | Chief Executive Officer at BNA | UK | Neuroscience and Society |
| Elisabeth Hildt | Biochemistry + Bioethics | PhD | Head of Research Group in Neuroethics, Philosophy Department, University of Mainz | Medical Ethicist | Germany | Enhancement |
| Emily Bell | Psychiatry | PhD | McGill University, Institute for Clinical Research | Neuroethics | Canada | Medical Research, Brain Death, Brain Stimulation, Neurosurgery, Psychiatric and Neurodegenerative diseases, Neuroscienc and Society |
| Eric Racine | Applied human sciences | PhD | McGill University, Institute for Clinical Research | Neuroethics | Canada | Medical Research, Addiction, Brain Death, Brain Stimulation, Enhancement, Legal Studies, Neuroimaging, Neurosurgery, Philosophy of Mind and Consciousness; Psychiatric and neurodegenerative diseases, Neuroscience and Society, Psychopharmacology |
| Ernst Feher | Economics | PhD | Global Distinguished Professor in Economics at New York University | Director of the UBS International Center of Economics in Society at the | Switzerland | Social Neuroscience |
| Erwin J Kompanje | Medical Doctor, Medical Ethics | MD + PhD | Department of Intensive Care, Erasmus MC University Medical Center Rotterdam | Neurology | The Netherlands | Brain Death |
| Fabien Perrin | ? | ? | Université de Lyon | ? | France | Brain Computer Interfaces |
| Fabio Formaglio | Medical Doctor | MD | Pain Medicine Center, Scientific Institute and Hospital San Raffaele, Milano | Neurology | Italy | Brain Death, Philosophy of Mind and Consciousness |
| Fabrice Jotterand | Theology + Bioethics | PhD | Institute for Biomedical Ethics, Universität Basel | Senior Researcher | Switzerland | Psychopharmacology |
| Farid F Youssef | Medical Doctor | PhD | Department of Pre-Clinical Sciences, Faculty of Medical Sciences, University of the West Indies | Lecturer | Trinidad and Tobago | Brain Computer Interfaces |
| Fiery Cushman | Cognitive Psychology | PhD | Assistant Professor, Department of Psychology, Harvard University | Moral Psychology Research Lab | US | Moral Theory |
| Frank Krüger | Cognitive Psychology | PhD | National Institute of Neurological Disorders and Stroke, National Institutes of Health, Bethesda | ? | US | Neuroimaging |
| Franklin Miller | Philosophy | PhD | National Institutes of Health, Department of Bioethics | Public Health | US | Medical Research, Brain Death, Neurosurgery |
| G J Wang | ? | PhD | Bookhaven Natoinal Laboratory | Addiction neuroscience | US | Addiction |
| Garry J. Bryson | Psychology | PhD | Yale University School of Medicine | Psychologist and associate research scientist | US | Psychiatric and neurodegenerative diseases |
| Geena Sankoorikal | ? | ? | Center for Cognitive Neuroscience, University of Pennsylvania | ? | US | Enhancement |
| Georg Northoff | Medical Doctor + Philosopher | MD + 2 PhD | Mind, Brain Imaging and Neuroethics Research Unit, University of Ottawa Institute of Mental Health Research | Neurophilosophy | Canada | Medical Research, Moral Theory |
| George Ainslie | Medical Doctor | MD + PhD | Department of Veterans Affairs Medical Center, Coatesville, PA and School of Economics, University of Cape Town, South Africa | Professor of Economics | Us + South Africa | Social Neuroscience |
| Grant R Gillett | Medical Doctor | MD + PhD | University of Otago, Division of Health Sciences | Bioethics | New Zealand | Brain Death, Neurosurgery |
| Gregory L. Belenky | Medical Doctor | MD | Walter Reed Army Institute of Research | ? | US | Psychopharmacology |
| Guglielmo Tamburrini | Philosophy | PhD | Department of Philosophy, Università die Napoli, Federico II | Professor of Philosophy of Science | Itlay | Brain Computer Interfaces |
| Gustave Moonen | Medical Doctor | MD + PhD | Department of Neurology, Université de Liège | Neurology | Belgium | Brain Death |
| Guy Kahane | Philosophy | PhD | Deputy Director and Research Fellow, Oxford Centre for Neuroethics, University of Oxford | Resarch Fellow | UK | Neuroimaging |
| Guy McKhann | Medical Doctor | MD, PhD | Department of Neurosurgery, Columbia University Medical School | Florence Irving Associate Professor of Neurological Surgery | US | Molecular Neurobiology and Genetics |
| Hanfried Helmchen | Medical Doctor | MD + PhD | Charité - Universitätsmedizin Berlin, Clinic of Psychiatry and Psychotherapy | Professor emeritus | Germany | Neuroscience and Society |
| Hans Förstl | Medical Doctor | MD + PhD | Direktor der Klinik und Poliklinik für Psychiatrie und Psychotherapie des Klinikums rechts der Isar, TU München | Professor of Psychiatry and Psychotherapy | Germany | Enhancement, Molecular Neurobiology and Genetics, Psychiatric and Neurodegenerative Diseases, Pschopharmacology |
| Helen S Mayberg | Medical Doctor | MD | Department of Psychiatry and Behavioural Science, Emory School of Medicine, Atlanta | Psychiatry, Neurology, and Radiology | US | Brain Stimulation, Social Neurosience |
| Henry T Greely | Law | JD | Stanford Law School | Law and neuroethics | US | Legal Studies, Neurosurgery |
| Hideaki Koizumi | ?? | PhD | Advanced Research Laboratory, Hitachi Ltd | Senior Chief Scientist of Hitachi's Research & Development Group | Japan | Neuroscience and Society |
| Hubert Doucet | Religious Studies | PhD | Universite de Montreal | Bioethics | Canada | Molecular Neurobiology and Genetics |
| Hubert H Fernandez | Medical Doctor | MD | Center for Neurological Restoration, Cleveland Clinic Lerner College of Medicine, Case Western Reserve University | Professor of Medicine (Neurology) | US | Brain Stimulation, Neurosurgery |
| Ineke Bolt | Philosophy | PhD | Ethics Institute of the Department of Philosophy of Utrecht University | Assistant Professor of Philosophy | The Netherlands | Enhancement, Psychopharmacology |
| Ivar Mendez | Medical Doctors | MD + PhD | Chairman of the Department of Surgery, University of Saskatchewan | Fred H. Wigmore Professor of Surgery | Canada | Neurosurgery |
| Ivenei E. Bramati | Physics + Economics | Msc + MBA | Cognitive and Behavioral Neuroscience Unit, labs-D'Or Hospital Network, Rio de Janeiro | PhD Candidate | Brazil | Social Neuroscience |
| J H Stephen | ? | ? | Department of Neurosurgery, Hospital of the University of Pennsylvania, Philadelphia | Neurosurgery | US | Addiction |
| Jaak Panksepp | Psychology and Neuroscience | PhD | Integrative Physiology and Neuroscience (IPN), Washington State University | Baily Endowed Chair of Animal Well-Being Science | US | Social Neuroscience |
| James Giordano | Biochemistry + Bioethics | PhD | Division of Integrative Physiology, Department of Biochemistry, Interdisciplinary Program in Neuroscience, Georgetown University, Washington, DC | Chief of the Neuroethics Studies Program in the Pellegrino Center for Clinical Bioethics, | US | Philosophy of Mind and Consciousness |
| James L Bernat | Medical Doctor | MD | Geisel School of Medicine, Dartmouth College | Neurology | US | Brain Death, Legal Studies, Philosophy of Mind and Consciousness |
| Jay Ingram | Microbiology | MSc | CTV Inc. | Retired Journalist | Canada | Neuroscience and Society |
| Jayne C. Lucke | Social Psychology | PhD | Faculty of Health Sciences, Australian Research Centre in Sex, Health and Society, Löa Trobe University Melbourne | Professor of Public Health, Director of the Australian Research Centre in Sex, Health & Society at La Trobe University | Australia | Psychopharmacology |
| Jean Decety | Psychology | PhD | Department of Psychology, The University of Chicago | Irving B. Harris Professor of Psychology | US | Moral Theory, Neuroimaging |
| Jefrey P Kahn | Medical Doctor | MD | Weill Medical College of Cornell University | Professor of Psychiatry | US | Molecular Neurobiology and Genetics, Neurosurgery |
| Jens Clausen | Biology, Philosophy | PhD | Department of Medical Ethics, University of Tübingen | Professor of Biomedical Ethics | Germany | Brain Computer Interfaces |
| Jeremy R. Gray | Cognitive Psychology | PhD | Department of Psychology, Michigan State University | Professor of Psychology | US | Neuroscience and Society |
| Jeremy Sugarman | Medical Doctor, Bioethics | MD, MPH, MA | Johns Hopkins Berman Institute of Bioethics, Johns Hopkins University, Baltimore | Harvey M. Meyerhoff Professor of Bioethics and Medicine | US | Molecular Neurobiology and Genetics |
| Jinger G Hoop | Medical Doctor | MD + MFA | Director of Research Ethics Consultation Service, University of Illinois at Chicago | Bioethics, Psychiatry | US | Medical Research, Psychiatric and neurodegenerative diseases |
| Joanne Kurtzberg | Medical Doctor | MD | Division of Pediatric Blood and Marrow Transplantation, Duke University Medical Center, Durham | Professor of Pediatrics and Pathology | US | Molecular / Gentics |
| John M. Darley | Social Psychology | PhD | Department of Psychology, Princeton University, Princeton | Warren Professor of Psychology | US | Social Neuroscience |
| Jonathan D. Cohen | Medical Doctor, Cognitive Psychology | MD + PhD | Department of Psychology, Center for the Study of Brain, Mind, and Behavior, Princeton University | Robert Bendheim and Lynn Bendheim Thoman Professor in Neuroscience | US | Moral Theory, Social Neuroscience |
| Jordan Grafman | Medical Doctor | MD + PhD | Cognitive Neurology and Alzheimer's Disease Center, Psychiatry and Behavioral Sciences, Northwestern University | Professor of Physical Medicine and Rehabilitation | US | Neuroimaging |
| Jörg Fegert | Medical Doctor | MD, PhD | Klinik für Kinder- und Jugendpsychiatrie, Universität Ulm | Director of University Hospital (Psychiatry) | Germany | Legal Studies |
| Jorge Moll | Medical Doctor | MD + PhD | Instituto D'Or de Pesquisa e Ensino | Behavorial and cognitive neurosciences | Brazil | Moral Theory, Social Neuroscience |
| Joseph J Fins | Medical Doctor | MD + PhD | Cornell University, Weill Medical College | Medical Ethics | US | Medical Research, Brain Death, Brain Stimulation, Neuroimaging; Philosophy of Mind and Consciousness, Social Neuroscience |
| Joseph T. Coyle | Medical Doctor | MD + PhD | Department of Psychiatry, Harvard Medical School, Belmont, Massachusetts | Eben S. Draper Chair of Psychiatry and Neuroscience | US | Molecular Neurobiology and Genetics |
| Joshua D Greene | Philosophy | PhD | Harvard, Department of Psychology | Social sciences | US | Moral Theory, Neuroimaging, Social Neuroscience |
| Judy Illes | Neuroscience | PhD | University British Columbia, Division of Neurology | Neurology | Canada | Medical Research, Addiction, Brain Death, Legal Studies, Moral Theory, Neuroimaging; Philosophy of Mind and Consciousness, Psychiatric and Neurodegenerative diseases, Neuroscience and Society |
| Julia Finkel | Medical Doctor | MD | Johns Hopkins Berman Institute of Bioethics, Johns Hopkins University, Baltimore | | US | Molecular Neurobiology and Genetics |
| Karin B Nelson | Medical Doctor | MD | National Institute of Neurological Disorders and Stroke, National Institutes of Health, Bethesda | Neurologist | US | Molecular /Genetics |
| Kate D Fenton | ? | ? | University of the West of England, Bristol | ? | UK | Neuroscience and Society |
| Kathrin Ohla | Psychology, Neuroscience | PhD | Department of Molecular Genetics, German Institute of Human Nutrition, Potsdam | Junior research group leader | Germany | Brain Computer Interfaces |
| Keith A. Harenski | Neuroscience | PhD | The Mind Research Network, Albuquerque | Assistant Professor of Translational Neuroscience | US | Moral Theory |
| Kelly D Foote | Medical Doctor | MD | Department of Neurosurgery, University of Florida | Neurosurgery | US | Brain Stimulation, Neurosurgery, Psychiatry and Neurodegenerative diseases |
| Kenneth R Forster | Physics | PhD | Department of Engeneering, University of Pennsylvania | Engeneering | US | Medical Research, |
| Kent A Kiehl | Psychology and Neuroscience | PhD | Department of Psychology, University of New Mexico | Psychology, Neuroscience and Law | US | Moral Theory, Neuroimaging |
| Kerri Smith | Journalist | ? | Nature | Editor of Natur Podcast | UK | Molecular Neurobiology and Genetics |
| Kevin D. Sauvé | Neuroscience | BSc | National Core for Neuroethics, The University of British Columbia, Vancouver | Graduate Student, Journalist | Canada | Neurosceience and Society |
| Kirschen, Matthew P | Medical Doctor | MD | Children's Hospital of Philadelphia, Division of Neurology | Pediatrician and Neurologist | US | Medical Research, |
| Klaus Lieb | Medical Doctor | MD | University Medical Centre, Mainz | Psychiatry | Germany | Addiction, Enhancement |
| L W Roberts | Medical Doctor + Philosopher | MD + MA | Department of Psychiatry and Behavioural Scioence, Stanford University | Psychiatry | US | Medical Research, Psychiatric and neurodegenerative diseases |
| Laura B Zahodne | Medical Doctor | MD + PhD | Cognitive Neuroscience Division, Department of Neurology, Columbia University College of Physicians and Surgeons | Associate Research Scientist | US | Neurosurgery |
| Laura B. Dunn | Medical Doctor | MD | UCSF School of Medicine, University of California, San Francisco | Professor of Psychiatry | US | Neuroscience and Society |
| Leigh E. Nystrom | Psychology | PhD | Scully Center for the Neuroscience of Mind and Behavior, Princeton Neuroscience Institute, Princeton University | Co-Director Neuroscience of Cognitive Control Laboratory | US | Moral Theory, Social Neuroscience |
| Liane L Young | Cognitive Psychology | PhD | Boston College, Department of Psychology | Morality | US | Legal Studies, Moral Theory, Neuroimaging, Philosophy of Mind and Consciousness, Social Neuroscience |
| Lindsey Kirsch-Darrow | ? | PhD | Division of Rehabilitation Psychology and Neuropsychology, Department of Physical, Medicine & Rehabilitation, The Johns Hopkins School of Medicine, Baltimore, MD | ? | US | Brain Stimulation, Neurosurgery |
| Luke Clarke | Psychology | PhD | Department of Psychology, University of Cambridge | Co-director of the Laboratory for Affect, Risk and Gambling Experiments | UK | Psychopharmacology |
| M L Kringelbach | Neuroscience | PhD | Aarhus Univ, Department of Clinical Medicine | Neuroscience | Denmark | Brain Stimulation, Neuroimaging |
| Mahendra Rao | Medical Doctor, Developmental Biology | MD + PhD | National Institute of Neurological Disorders and Stroke, National Institutes of Health, Bethesda | Vice President for Regenerative Medicine | US | Molecular Neurobiology and Genetics |
| Marc D Hauser | Psychology | PhD | Program in Neurosciences, Harvard University | Professor of Psychology and Program in Neurosciences, and Director of Primate Cognitive Neuroscience Laboratory | US | Moral Theory |
| Mark Bernstein | Neurosurgery | MD | University of Toronto, Department of Surgery | Research ethics | Canada | Medical Research, Neurosurgery, Psychiatric and Neurodegenerative Diseases |
| Martha Farah | Experimental Psychology | PhD | University of Pennsylvania, Center for Neuroscience and Society | Natural Sciences | US | Medical Research, Enhancement, Philosophy of Mind and Consciousness |
| Mary A. Kautz | ? | ? | Walter Reed Army Institute of Research | ? | US | Psychopharmacology |
| Matthew S. Shane | Neuroscience | PhD | The Mind Research Network, Albuquerque | Assistant Professor of Translational Neuroscience | US | Neuroimaging |
| Matthis Synofzik | Philosophy and Medicine | MD + MA | Tuebingen, Hertie Institute for Clinical Brain Research | Geriatric Rehabilitation | Germany | Moral Theory, Philosophy of Mind and Consciousness, Psychiatric and Neurodegenerative Diseases, Psychopharmacology |
| Michael A. Williams | Medical Doctor | MD | Sandra and Malcolm Berman Brain & Spine Institute, Department of Neurology, Sinai Hospital of Baltimore | Neurologist | US | Enhancement |
| Michael Greicius | Medical Doctor | MD | Department of Neurology and Neurological Sciences, Stanford University | Assistant Professor of Neurology | US | Neuroscience and Society |
| Michael Johnston | Medical Doctor | MD + PhD | Division of Neurology and Developmental Medicine, Kennedy Krieger Institute, Baltimore | Senior Vice President and Chief Medical Officer | US | Molecular Neurobiology and Genetics |
| Michael R. Matthews | Philosophy of Education | PhD | School of Education, University of New South Wales, Sydney | Honorary Associate Professor | Australia | Neuroscience and Society |
| Michael S Gazzaniga | Psychobiology | PhD | UCSD, Center for Cognitive Neuroscience | Cognitive neuroscience | US | Legal Studies, Neuroimaging, Philosophy of Mind and Consciousness |
| Michael S Okun | Medical Doctor | MD | Centre for Movement Disorders and Neurorestauration, University of Florida | Departments of Neurology, Neurosurgery, Neuroscience, Psychiatry and History | US | Brain Stimulation, Neurosurgery, Psychiatric and Nneurodegenerative diseases |
| Michael S. Pratte | Psychology | PhD | Perception and Neuroscience Lab, Vanderbilt University | Postdoctoral Research Fellow | US | Philosophy of Mind and Consciousness |
| Michaela Christman | Medical Doctor | MD | University Medical Centre, Mainz | Medical Doctor | Germany | Addiction, Enhancement |
| Morris David Bell | Medical Doctor | MD + PhD | Department of Psychiatry at Yale University School of Medicine | Professor of Psychiatry | US | Brain Computer Interfaces, Psychiatric and Neurodegenerative Diseases |
| Nancy Jo Wesenstein | ? | ? | Walter Reed Army Institute of Research, Division of Psychiatry and Neuroscience, Behavioural Biology | Neurobiology | US | Medical Research, Philosophy of Mind and Consciousness, Psychopharmacology |
| Neil Levy | Philosophy | PhD | Deputy Director of the Oxford Centre for Neuroethics, and Head of Neuroethics at the Florey Neuroscience Institutes, University of Melbourne | Professor of Neuroethics | UK / Australia | Enhancement, Moral Theory, Philosophy of Mind and Consciousness |
| Nicholas D Schiff | Medical Doctor | MD + PhD | Cornell University, Weill Medical College | Neuroscience | US | Brain Death, Philosophy of Mind and Consciousness |
| Nir Lipsman | Neurosurgery | MD | University of Toronto, Department of Surgery | Neurosurgery | Canada | Brain Stimulation, Neurosurgery, Psychiatric and Neurodegeneratibve Diseases |
| Nora D Volkow | Medical Doctor | MD + PhD | National Institute on Drug Abuse | Addiction neuroscience | US | Addiction |
| Nozomi Mizushima | ? | ? | Interfaculty Initiative in Information Studies, University of Tokyo, Tokyo | ? | Japan | Brain Computer Interfaces |
| O S Tenovuo | Medical Doctor | MD + PhD | Turku University Central Hospital | Neurotraumatology | Finland | Brain Death |
| Olaf Blanke | Medical Doctor | MD + PhD | Center for Neuroprosthetics, Bertarelli Foundation Chair in Cognitive Neuroprosthetics at Ecole Polytechnique Fédérale de Lausanne (EPFL) | Professor of Neuroscience | Switzerland | Legal Studies, philosophy of Mind and Consciousness |
| Oliver R Goodenough | Law | JD | Vermont Law School | Professor of Law | US | Legal Studies |
| Orfek Bar-Ilhan | Biology | MSc | The Stanford Center for Biomedical Ethics | ? | US | Neuroscience and Society |
| Osamu Sakura | ? | PhD | University of Tokyo, Interdisciplinary Information Studies | Cultural and Human Information Studies | Japan | Medical Research, Legal Studies, Neuroscience and Society, Social Neuroscience |
| P H Sonninen | ? | ? | Turku University Central Hospital | ? | Finland | Brain Death |
| Paul A. Howard-Jones | ? | PhD | Graduate School of Education, University of Bristol | Reader in Neuroscience and Education | UK | Neuroscience and Society |
| Paul E Holtzheimer | Medical Doctor; Clinical Research | MD + M.S. | Geisel School of Medicine at Dartmouth | Professor of Psychiatry | US | Brain Stimulation |
| Paul Ford | Philosophy | PhD | Cleveland Clinic, Center for Ethics, Humanities and Spiritual Care | neuroethics | US | Moral Theory, Neurosurgery |
| Paul M. Thompson | Mathematics, Classics, Neuroscinece | PhD | Laboratory of Neuroimaging, UCLA School of Medicine, Los Angeles | Professor of Neurology and Biomedical Engineering | US | Neuroscience and Society |
| Paul S Applebaum | Medical Doctor | MD | Division of Psychiatry, Law, and Ethics, Department of Psychiatry, Columbia University | Psychiatry | US | Medical Research, Neuroscience and Society |
| Peter B Reiner | Neuroscience | PhD | National Core for Neuroethics at the University of British Columbia | Neuroethics | Canada | Addiction, Neuroscience and Society |
| Peter McKnight | Law, Jounalism, Philosophy, Psychology | ? | Columnist, Vancouver Sun, Vancouver | Adjunct professor of Criminology at Simon Fraser University | Canada | Neuroscience and Society |
| Ramon L Rodriguez | Medical Doctor | MD | Centre for Movement Disorders and Neurorestauration, University of Florida | Associate Professor of Neurology | US | Brain Stimulation, Neuroisurgery |
| Rebecca Saxe | Cognitive neuroscience | PhD | MIT, Department of Brain and Cognitive Sciences | Cognitive neuroscience | US | Moral Theory, Neuroimaging, Philosophy of Mind and Consciousness |
| Ricardo De Oliveira-Souza | Neurological Sciences | MD + PhD | Instituto D'Or de Pesquisa e Ensino | Neurologie | Brasil | Moral Theory, Social Neuroscience |
| Robert H. Blank | Political Science | PhD | Department of Political Science, University of Canterbury, New Zealand | Professor of Political Science | New Zealand | Neuroscience and Society |
| Robert Perneczky | Medical Doctor | MD + PhD | Faculty of Medicine, School of Public Health, Imperial College, London | Reader in Cognitive Impairment and Dementia | UK | Psychiatric and neurodegenerative diseases |
| Roger Barker | Medical Doctor | MD + PhD | Cambridge University, Department of Clinical Neurosciences | therapies for neurodegenerative diseases | UK | Molecular Neurobiology and Genetics |
| Roland H. Grabner | Psychology | PhD | Institute for Behavioral Sciences, Swiss Federal Institute of Technology (ETH), Zürich | Professor of Psychology | Switzerland | Neuroscience and Society |
| Roland Zahn | Medical Doctor | MD + PhD | Centre for Affective Disorders at the Institute of Psychiatry, King's College London | Senior Clinical Lecturer (Mood Disorders) | UK | Neuroimaging |
| S Honeybul | Medical Doctor | MD | Department of Neurosurgery, Sir Charles Gairdner Hospital and Royal Perth Hospital, Perth | Neurosurgery | Australia | Brain Death |
| S L Owen | ? | D.Phil | Centre for Simulation in Healthcare, University of Portsmouth | ? | UK | Brain Stimulation, Neuroimaging |
| Samuel Weiss | Medical Doctor | MD + PhD | Hotchkiss Brain Institute, Health Research Innovation Centre, University of Calgary | Director | Canada | Neuroscience and Society |
| Sandra Blakeslee | Political Science | MSc | Independent Science Writer and Journalist | Independent Science Writer and Journalist | US | Neuroscience and Society |
| Sidney Bloch | Medical Doctor | MD | Department of Psychiatry University of Melbourne | Psychiatry | Australia | Medical Research, |
| Simon M Outram | Cultural Anthropology, Science and Technology | PhD | Institute of Sports, Exercise and Active Living, Victoria University, Melbourne | Research Fellow | Australia | Enhancement, Psychopharmacology |
| Sofia Lombera | Biomedicine, Bioscience and Society | MSc | National Core for Neuroethics at University of British Columbia | Research and Global Partnerships Manager | Canada | Neuroscience and Society |
| Stacey A Tovino | Law | JD + PhD | Drake University Law School | Associate Professor of Law | US | Legal Studies |
| Stellan Welin | Philosophy | PhD | Division of Health and Society, Department of Medical and Helath Sciences, Linköping University | Professor of Bioethics | Sweden | Philosophy of Mind and Consciousness, Social Neuroscience |
| Stephan Schleim | Cognitive Science, Philosophy | PhD | University of Groningen | Assistant Professor of History of Psychology | The Netherlands | Legal Studies, Moral Theory |
| Stephen J Morse | Law | JD + PhD | University of Pennsylvania, Law School | Psychology and Law in Psychiatry | US | Legal Studies |
| Steven Laureys | Medical Doctor | MD | Sart Tilman Liège University Hospital, Department of Neurology | End-of-life medicine | Belgium | Brain Death, Philosophy of Mind and Consciousness |
| Tamami Fukushi | Behavioral science | PhD | Japan Science and Technology Agency, Center for Research on Brain-Science&Society | Neuroethics and Neurophysiology | Japan | Neuroscience and Society, Social Neuroscience |
| Tania Singer | Psychology | PhD | Max Planck Institute for Human Cognitive and Brain Sciences, Leipzig | Director, Department of Social Neuroscience | Germany | Social Neuroscience |
| Thomas A Raffin | Medical Doctor | MD | Department of Medicine, Stanford University | Medicine and Biomedical Ethics | US | Addiction |
| Thomas E Schlaepfer | Medical Doctor | PhD | Department of Psychiatry and Psychotherapy, University Hospital Bonn | Professor of Psychiatry | Germany | Brain Computer Interfaces, Brain Stimulation, Psychiatric and Neurodegerative diseases |
| Thomas J. Balkin | Experimental Psychology | PhD | Walter Reed Army Institute of Research, Division of Psychiatry and Neuroscience, Behavioural Biology | Neurobiology | US | Medical Research, Philosophy of Mind of consciousness, Psychopharmacology |
| Thomas Nadelhoffer | Philosophy | PhD | College of Charleston, Department of Philosophy | assistant professor of philosophy | US | Legal Studies |
| Thorsten Galert | Chemistry, Philosophy | PhD | Deutsches Referenzzentrum für Ethik in den Biowissenschaften | Mitarbeiter der wissenschaftlichen Abteilung | Germany | Brain Computer Interfaces |
| Tipu Aziz | Neuroscience | PhD | University of Oxford, Academic Department of Surgery | Neurosurgery | UK | Brain Stimulation, Neuroimaging, Neurosurgery |
| Trevor W Robbins | Experimental Psychology | PhD | Department of Psychology, Cambridge University | Neuroscience | UK | Medical Research, Addiction, Psychopharmacology |
| Walter Glannon | Philosophy | PhD | Calgary University, Department of Philosophy and Faculty of Medicine | Biomedical ethics | Canada | Addiction, Psychiatric and Neurodegenerative Diseases, Psychopharmacology |
| Walter Sinnott-Armstrong | Philosophy | PhD | Department of Philosophy, Duke University | Professor of Practical Ethics | US | Legal Studies, Neuroimaging |
| Wayne D. Hall | ? | PhD | University of Queensland, UQ center for clinical research | Addiction neuroethics | Australia | Addiction, Enhancement, Legal Studies, Psychopharmacology |
| Wise Young | Neuroscience, Molecular Biology | PhD | WM Keck Center for Collaborative Neuroscience, Rutgers University | The Richard H. Shinell Chair In Neuroscience | US | Molecular Neurobiology and Genetics, Neurosurgery |
| Yuko Akitsuki | Medical Doctor | MD | Department of Functional Imaging, Institute of Development, Aging and Cancer (IDAC), Tohoku University | Assistant Professor | Japan | Social Neuroscience |

**Supplement 4**

**Comparison of methods connecting papers to subject-categories by a defined set of keywords:** A constant number of keywords as applied in method II overall connects a significantly lower number of papers to the respective subject-categories. The specificity of the keywords used in method I is more effective to connect papers to the correct category, even though the number of keywords per subject-category is not constant. A high specificity of keywords trumps a constant distribution of keywords.

|  | Number of keywords, method I | Number of keywords, method II | Number of papers, method I | Number of papers, method II | (Nb. papers I) – (Nb. papers II) |
| --- | --- | --- | --- | --- | --- |
| Addiction | 9 | 16 | 55 | 92 | 37 |
| Brain Death / Severe disorders of consciousness | 23 | 16 | 204 | 129 | -75 |
| Brain stimulation | 6 | 16 | 154 | 201 | 47 |
| Enhancement | 14 | 16 | 149 | 88 | -61 |
| Legal Studies | 9 | 16 | 267 | 229 | -38 |
| Molecular and genetic neuroscience | 5 | 16 | 61 | 114 | 53 |
| Moral Theory | 26 | 16 | 346 | 352 | 6 |
| Medical Research and Medicine | 13 | 16 | 326 | 234 | -92 |
| Neuroimaging | 11 | 16 | 477 | 308 | -169 |
| Neuroscience and society | 18 | 16 | 237 | 209 | -28 |
| Neurosurgery | 11 | 16 | 268 | 119 | -149 |
| Philosophy of mind and consciousness | 27 | 16 | 333 | 336 | 3 |
| Psychiatric and neurodegenerative diseases and disorders | 27 | 16 | 632 | 379 | -253 |
| Psychopharmacology | 18 | 16 | 223 | 85 | -138 |
| Social and Economic neuroscience | 27 | 16 | 205 | 126 | -79 |
|  |  |  |  |  |  |
|  |  | TOTAL: | 3937 | 3001 |  |
