# Supplementary material for: Neuroethics 1995–2012. A Bibliometric Analysis of the Guiding Themes of an Emerging Research Field
Source: Front Hum Neurosci. 2016 Jul 1;10:336. doi: 10.3389/fnhum.2016.00336 (PMC4929847; doi:10.3389/fnhum.2016.00336)
Supplement: Supplementary file 4 [file Supplement5.DOCX]

**Supplement 5**

**Comparison of applied and alternative method of keyword-based publication retrieval**

| **Subject-Category** | **Number of keywords, method used in the paper** | **Number of keywords, alternative method** | **Number of papers, method used in the paper** | **Number of papers, alternative method** | **diff** |
| --- | --- | --- | --- | --- | --- |
| Addiction | 9 | 16 | 55 | 92 | 37 |
| Brain Death / Sever disorders of consciousness | 23 | 16 | 204 | 129 | -75 |
| Brain stimulation | 6 | 16 | 154 | 201 | 47 |
| Enhancement | 14 | 16 | 149 | 88 | -61 |
| Legal Studies | 9 | 16 | 267 | 229 | -38 |
| Medical Research and Medicine | 13 | 16 | 326 | 234 | -92 |
| Molecular / Genetics | 5 | 16 | 61 | 114 | 53 |
| Moral Theory | 26 | 16 | 346 | 352 | 6 |
| Neuroimaging | 11 | 16 | 477 | 308 | -169 |
| Neuroscience and society | 18 | 16 | 237 | 209 | -28 |
| Neurosurgery | 11 | 16 | 268 | 119 | -149 |
| Philosophy of Mind and Consciousness | 27 | 16 | 333 | 336 | 3 |
| Psychiatric and neurodegenerative diseases and disorders | 27 | 16 | 632 | 379 | -253 |
| Psychopharmacology | 18 | 16 | 223 | 85 | -138 |
| Social and Econ neuroscience | 27 | 16 | 205 | 126 | -79 |
|  |  |  |  |  |  |
|  |  | **TOTAL:** | 3937 | 3001 |  |
